# Study of the underlying event in top quark pair production in $\mathrm {p}\mathrm {p}$ collisions at 13$~\text {Te}\text {V}$

**DOI:** 10.1140/epjc/s10052-019-6620-z

**Published:** 2019-02-07

**Authors:** A. M. Sirunyan, A. Tumasyan, W. Adam, F. Ambrogi, E. Asilar, T. Bergauer, J. Brandstetter, E. Brondolin, M. Dragicevic, J. Erö, A. Escalante Del Valle, M. Flechl, R. Frühwirth, V. M. Ghete, J. Hrubec, M. Jeitler, N. Krammer, I. Krätschmer, D. Liko, T. Madlener, I. Mikulec, N. Rad, H. Rohringer, J. Schieck, R. Schöfbeck, M. Spanring, D. Spitzbart, A. Taurok, W. Waltenberger, J. Wittmann, C.-E. Wulz, M. Zarucki, V. Chekhovsky, V. Mossolov, J. Suarez Gonzalez, E. A. De Wolf, D. Di Croce, X. Janssen, J. Lauwers, M. Pieters, M. Van De Klundert, H. Van Haevermaet, P. Van Mechelen, N. Van Remortel, S. Abu Zeid, F. Blekman, J. D’Hondt, I. De Bruyn, J. De Clercq, K. Deroover, G. Flouris, D. Lontkovskyi, S. Lowette, I. Marchesini, S. Moortgat, L. Moreels, Q. Python, K. Skovpen, S. Tavernier, W. Van Doninck, P. Van Mulders, I. Van Parijs, D. Beghin, B. Bilin, H. Brun, B. Clerbaux, G. De Lentdecker, H. Delannoy, B. Dorney, G. Fasanella, L. Favart, R. Goldouzian, A. Grebenyuk, A. K. Kalsi, T. Lenzi, J. Luetic, N. Postiau, E. Starling, L. Thomas, C. Vander Velde, P. Vanlaer, D. Vannerom, Q. Wang, T. Cornelis, D. Dobur, A. Fagot, M. Gul, I. Khvastunov, D. Poyraz, C. Roskas, D. Trocino, M. Tytgat, W. Verbeke, B. Vermassen, M. Vit, N. Zaganidis, H. Bakhshiansohi, O. Bondu, S. Brochet, G. Bruno, P. David, C. Delaere, M. Delcourt, B. Francois, A. Giammanco, G. Krintiras, V. Lemaitre, A. Magitteri, A. Mertens, M. Musich, K. Piotrzkowski, A. Saggio, M. Vidal Marono, S. Wertz, J. Zobec, F. L. Alves, G. A. Alves, L. Brito, G. Correia Silva, C. Hensel, A. Moraes, M. E. Pol, P. Rebello Teles, E. Belchior Batista Das Chagas, W. Carvalho, J. Chinellato, E. Coelho, E. M. Da Costa, G. G. Da Silveira, D. De Jesus Damiao, C. De Oliveira Martins, S. Fonseca De Souza, H. Malbouisson, D. Matos Figueiredo, M. Melo De Almeida, C. Mora Herrera, L. Mundim, H. Nogima, W. L. Prado Da Silva, L. J. Sanchez Rosas, A. Santoro, A. Sznajder, M. Thiel, E. J. Tonelli Manganote, F. Torres Da Silva De Araujo, A. Vilela Pereira, S. Ahuja, C. A. Bernardes, L. Calligaris, T. R. Fernandez Perez Tomei, E. M. Gregores, P. G. Mercadante, S. F. Novaes, SandraS. Padula, D. Romero Abad, A. Aleksandrov, R. Hadjiiska, P. Iaydjiev, A. Marinov, M. Misheva, M. Rodozov, M. Shopova, G. Sultanov, A. Dimitrov, L. Litov, B. Pavlov, P. Petkov, W. Fang, X. Gao, L. Yuan, M. Ahmad, J. G. Bian, G. M. Chen, H. S. Chen, M. Chen, Y. Chen, C. H. Jiang, D. Leggat, H. Liao, Z. Liu, F. Romeo, S. M. Shaheen, A. Spiezia, J. Tao, C. Wang, Z. Wang, E. Yazgan, H. Zhang, J. Zhao, Y. Ban, G. Chen, A. Levin, J. Li, L. Li, Q. Li, Y. Mao, S. J. Qian, D. Wang, Z. Xu, Y. Wang, C. Avila, A. Cabrera, C. A. Carrillo Montoya, L. F. Chaparro Sierra, C. Florez, C. F. González Hernández, M. A. Segura Delgado, B. Courbon, N. Godinovic, D. Lelas, I. Puljak, T. Sculac, Z. Antunovic, M. Kovac, V. Brigljevic, D. Ferencek, K. Kadija, B. Mesic, A. Starodumov, T. Susa, M. W. Ather, A. Attikis, G. Mavromanolakis, J. Mousa, C. Nicolaou, F. Ptochos, P. A. Razis, H. Rykaczewski, M. Finger, M. Finger, E. Ayala, E. Carrera Jarrin, H. Abdalla, A. A. Abdelalim, A. Mohamed, A. Carvalho Antunes De Oliveira, R. K. Dewanjee, K. Ehataht, M. Kadastik, M. Raidal, C. Veelken, P. Eerola, H. Kirschenmann, J. Pekkanen, M. Voutilainen, J. Havukainen, J. K. Heikkilä, T. Järvinen, V. Karimäki, R. Kinnunen, T. Lampén, K. Lassila-Perini, S. Laurila, S. Lehti, T. Lindén, P. Luukka, T. Mäenpää, H. Siikonen, E. Tuominen, J. Tuominiemi, T. Tuuva, M. Besancon, F. Couderc, M. Dejardin, D. Denegri, J. L. Faure, F. Ferri, S. Ganjour, A. Givernaud, P. Gras, G. Hamel de Monchenault, P. Jarry, C. Leloup, E. Locci, J. Malcles, G. Negro, J. Rander, A. Rosowsky, M. Ö. Sahin, M. Titov, A. Abdulsalam, C. Amendola, I. Antropov, F. Beaudette, P. Busson, C. Charlot, R. Granier de Cassagnac, I. Kucher, S. Lisniak, A. Lobanov, J. Martin Perez, M. Nguyen, C. Ochando, G. Ortona, P. Pigard, R. Salerno, J. B. Sauvan, Y. Sirois, A. G. Stahl Leiton, A. Zabi, A. Zghiche, J.-L. Agram, J. Andrea, D. Bloch, J.-M. Brom, E. C. Chabert, V Cherepanov, C. Collard, E. Conte, J.-C. Fontaine, D. Gelé, U. Goerlach, M. Jansová, A.-C. Le Bihan, N. Tonon, P. Van Hove, S. Gadrat, S. Beauceron, C. Bernet, G. Boudoul, N. Chanon, R. Chierici, D. Contardo, P. Depasse, H. El Mamouni, J. Fay, L. Finco, S. Gascon, M. Gouzevitch, G. Grenier, B. Ille, F. Lagarde, I. B. Laktineh, H. Lattaud, M. Lethuillier, L. Mirabito, A. L. Pequegnot, S. Perries, A. Popov, V. Sordini, M. Vander Donckt, S. Viret, S. Zhang, A. Khvedelidze, D. Lomidze, C. Autermann, L. Feld, M. K. Kiesel, K. Klein, M. Lipinski, M. Preuten, M. P. Rauch, C. Schomakers, J. Schulz, M. Teroerde, B. Wittmer, V. Zhukov, A. Albert, D. Duchardt, M. Endres, M. Erdmann, T. Esch, R. Fischer, S. Ghosh, A. Güth, T. Hebbeker, C. Heidemann, K. Hoepfner, H. Keller, S. Knutzen, L. Mastrolorenzo, M. Merschmeyer, A. Meyer, P. Millet, S. Mukherjee, T. Pook, M. Radziej, H. Reithler, M. Rieger, F. Scheuch, A. Schmidt, D. Teyssier, G. Flügge, O. Hlushchenko, B. Kargoll, T. Kress, A. Künsken, T. Müller, A. Nehrkorn, A. Nowack, C. Pistone, O. Pooth, H. Sert, A. Stahl, M. Aldaya Martin, T. Arndt, C. Asawatangtrakuldee, I. Babounikau, K. Beernaert, O. Behnke, U. Behrens, A. Bermúdez Martínez, D. Bertsche, A. A. Bin Anuar, K. Borras, V. Botta, A. Campbell, P. Connor, C. Contreras-Campana, F. Costanza, V. Danilov, A. De Wit, M. M. Defranchis, C. Diez Pardos, D. Domínguez Damiani, G. Eckerlin, T. Eichhorn, A. Elwood, E. Eren, E. Gallo, A. Geiser, J. M. Grados Luyando, A. Grohsjean, P. Gunnellini, M. Guthoff, M. Haranko, A. Harb, J. Hauk, H. Jung, M. Kasemann, J. Keaveney, C. Kleinwort, J. Knolle, D. Krücker, W. Lange, A. Lelek, T. Lenz, K. Lipka, W. Lohmann, R. Mankel, I.-A. Melzer-Pellmann, A. B. Meyer, M. Meyer, M. Missiroli, G. Mittag, J. Mnich, V. Myronenko, S. K. Pflitsch, D. Pitzl, A. Raspereza, M. Savitskyi, P. Saxena, P. Schütze, C. Schwanenberger, R. Shevchenko, A. Singh, N. Stefaniuk, H. Tholen, A. Vagnerini, G. P. Van Onsem, R. Walsh, Y. Wen, K. Wichmann, C. Wissing, O. Zenaiev, R. Aggleton, S. Bein, L. Benato, A. Benecke, V. Blobel, M. Centis Vignali, T. Dreyer, E. Garutti, D. Gonzalez, J. Haller, A. Hinzmann, A. Karavdina, G. Kasieczka, R. Klanner, R. Kogler, N. Kovalchuk, S. Kurz, V. Kutzner, J. Lange, D. Marconi, J. Multhaup, M. Niedziela, D. Nowatschin, A. Perieanu, A. Reimers, O. Rieger, C. Scharf, P. Schleper, S. Schumann, J. Schwandt, J. Sonneveld, H. Stadie, G. Steinbrück, F. M. Stober, M. Stöver, D. Troendle, A. Vanhoefer, B. Vormwald, M. Akbiyik, C. Barth, M. Baselga, S. Baur, E. Butz, R. Caspart, T. Chwalek, F. Colombo, W. De Boer, A. Dierlamm, N. Faltermann, B. Freund, M. Giffels, M. A. Harrendorf, F. Hartmann, S. M. Heindl, U. Husemann, F. Kassel, I. Katkov, S. Kudella, H. Mildner, S. Mitra, M. U. Mozer, Th. Müller, M. Plagge, G. Quast, K. Rabbertz, M. Schröder, I. Shvetsov, G. Sieber, H. J. Simonis, R. Ulrich, S. Wayand, M. Weber, T. Weiler, S. Williamson, C. Wöhrmann, R. Wolf, G. Anagnostou, G. Daskalakis, T. Geralis, A. Kyriakis, D. Loukas, G. Paspalaki, I. Topsis-Giotis, G. Karathanasis, S. Kesisoglou, P. Kontaxakis, A. Panagiotou, N. Saoulidou, E. Tziaferi, K. Vellidis, K. Kousouris, I. Papakrivopoulos, G. Tsipolitis, I. Evangelou, C. Foudas, P. Gianneios, P. Katsoulis, P. Kokkas, S. Mallios, N. Manthos, I. Papadopoulos, E. Paradas, J. Strologas, F. A. Triantis, D. Tsitsonis, M. Bartók, M. Csanad, N. Filipovic, P. Major, M. I. Nagy, G. Pasztor, O. Surányi, G. I. Veres, G. Bencze, C. Hajdu, D. Horvath, Á. Hunyadi, F. Sikler, T. Á. Vámi, V. Veszpremi, G. Vesztergombi, N. Beni, S. Czellar, J. Karancsi, A. Makovec, J. Molnar, Z. Szillasi, P. Raics, Z. L. Trocsanyi, B. Ujvari, S. Choudhury, J. R. Komaragiri, P. C. Tiwari, S. Bahinipati, C. Kar, P. Mal, K. Mandal, A. Nayak, D. K. Sahoo, S. K. Swain, S. Bansal, S. B. Beri, V. Bhatnagar, S. Chauhan, R. Chawla, N. Dhingra, R. Gupta, A. Kaur, A. Kaur, M. Kaur, S. Kaur, R. Kumar, P. Kumari, M. Lohan, A. Mehta, K. Sandeep, S. Sharma, J. B. Singh, G. Walia, A. Bhardwaj, B. C. Choudhary, R. B. Garg, M. Gola, S. Keshri, Ashok Kumar, S. Malhotra, M. Naimuddin, P. Priyanka, K. Ranjan, Aashaq Shah, R. Sharma, R. Bhardwaj, M. Bharti, R. Bhattacharya, S. Bhattacharya, U. Bhawandeep, D. Bhowmik, S. Dey, S. Dutt, S. Dutta, S. Ghosh, K. Mondal, S. Nandan, A. Purohit, P. K. Rout, A. Roy, S. Roy Chowdhury, S. Sarkar, M. Sharan, B. Singh, S. Thakur, P. K. Behera, R. Chudasama, D. Dutta, V. Jha, V. Kumar, P. K. Netrakanti, L. M. Pant, P. Shukla, T. Aziz, M. A. Bhat, S. Dugad, G. B. Mohanty, N. Sur, B. Sutar, RavindraKumar Verma, S. Banerjee, S. Bhattacharya, S. Chatterjee, P. Das, M. Guchait, Sa. Jain, S. Kumar, M. Maity, G. Majumder, K. Mazumdar, N. Sahoo, T. Sarkar, S. Chauhan, S. Dube, V. Hegde, A. Kapoor, K. Kothekar, S. Pandey, A. Rane, S. Sharma, S. Chenarani, E. Eskandari Tadavani, S. M. Etesami, M. Khakzad, M. Mohammadi Najafabadi, M. Naseri, F. Rezaei Hosseinabadi, B. Safarzadeh, M. Zeinali, M. Felcini, M. Grunewald, M. Abbrescia, C. Calabria, A. Colaleo, D. Creanza, L. Cristella, N. De Filippis, M. De Palma, A. Di Florio, F. Errico, L. Fiore, A. Gelmi, G. Iaselli, S. Lezki, G. Maggi, M. Maggi, G. Miniello, S. My, S. Nuzzo, A. Pompili, G. Pugliese, R. Radogna, A. Ranieri, A. Sharma, L. Silvestris, R. Venditti, P. Verwilligen, G. Zito, G. Abbiendi, C. Battilana, D. Bonacorsi, L. Borgonovi, S. Braibant-Giacomelli, R. Campanini, P. Capiluppi, A. Castro, F. R. Cavallo, S. S. Chhibra, C. Ciocca, G. Codispoti, M. Cuffiani, G. M. Dallavalle, F. Fabbri, A. Fanfani, P. Giacomelli, C. Grandi, L. Guiducci, F. Iemmi, S. Marcellini, G. Masetti, A. Montanari, F. L. Navarria, A. Perrotta, F. Primavera, A. M. Rossi, T. Rovelli, G. P. Siroli, N. Tosi, S. Albergo, A. Di Mattia, R. Potenza, A. Tricomi, C. Tuve, G. Barbagli, K. Chatterjee, V. Ciulli, C. Civinini, R. D’Alessandro, E. Focardi, G. Latino, P. Lenzi, M. Meschini, S. Paoletti, L. Russo, G. Sguazzoni, D. Strom, L. Viliani, L. Benussi, S. Bianco, F. Fabbri, D. Piccolo, F. Ferro, F. Ravera, E. Robutti, S. Tosi, A. Benaglia, A. Beschi, L. Brianza, F. Brivio, V. Ciriolo, S. Di Guida, M. E. Dinardo, S. Fiorendi, S. Gennai, A. Ghezzi, P. Govoni, M. Malberti, S. Malvezzi, A. Massironi, D. Menasce, L. Moroni, M. Paganoni, D. Pedrini, S. Ragazzi, T. Tabarelli de Fatis, S. Buontempo, N. Cavallo, A. Di Crescenzo, F. Fabozzi, F. Fienga, G. Galati, A. O. M. Iorio, W. A. Khan, L. Lista, S. Meola, P. Paolucci, C. Sciacca, E. Voevodina, P. Azzi, N. Bacchetta, D. Bisello, A. Boletti, A. Bragagnolo, R. Carlin, P. Checchia, M. Dall’Osso, P. De Castro Manzano, T. Dorigo, F. Gasparini, A. Gozzelino, S. Lacaprara, P. Lujan, M. Margoni, A. T. Meneguzzo, N. Pozzobon, P. Ronchese, R. Rossin, F. Simonetto, A. Tiko, E. Torassa, S. Ventura, M. Zanetti, P. Zotto, G. Zumerle, A. Braghieri, A. Magnani, P. Montagna, S. P. Ratti, V. Re, M. Ressegotti, C. Riccardi, P. Salvini, I. Vai, P. Vitulo, L. Alunni Solestizi, M. Biasini, G. M. Bilei, C. Cecchi, D. Ciangottini, L. Fanò, P. Lariccia, E. Manoni, G. Mantovani, V. Mariani, M. Menichelli, A. Rossi, A. Santocchia, D. Spiga, K. Androsov, P. Azzurri, G. Bagliesi, L. Bianchini, T. Boccali, L. Borrello, R. Castaldi, M. A. Ciocci, R. Dell’Orso, G. Fedi, L. Giannini, A. Giassi, M. T. Grippo, F. Ligabue, E. Manca, G. Mandorli, A. Messineo, F. Palla, A. Rizzi, P. Spagnolo, R. Tenchini, G. Tonelli, A. Venturi, P. G. Verdini, L. Barone, F. Cavallari, M. Cipriani, N. Daci, D. Del Re, E. Di Marco, M. Diemoz, S. Gelli, E. Longo, B. Marzocchi, P. Meridiani, G. Organtini, F. Pandolfi, R. Paramatti, F. Preiato, S. Rahatlou, C. Rovelli, F. Santanastasio, N. Amapane, R. Arcidiacono, S. Argiro, M. Arneodo, N. Bartosik, R. Bellan, C. Biino, N. Cartiglia, F. Cenna, S. Cometti, M. Costa, R. Covarelli, N. Demaria, B. Kiani, C. Mariotti, S. Maselli, E. Migliore, V. Monaco, E. Monteil, M. Monteno, M. M. Obertino, L. Pacher, N. Pastrone, M. Pelliccioni, G. L. Pinna Angioni, A. Romero, M. Ruspa, R. Sacchi, K. Shchelina, V. Sola, A. Solano, D. Soldi, A. Staiano, S. Belforte, V. Candelise, M. Casarsa, F. Cossutti, G. Della Ricca, F. Vazzoler, A. Zanetti, D. H. Kim, G. N. Kim, M. S. Kim, J. Lee, S. Lee, S. W. Lee, C. S. Moon, Y. D. Oh, S. Sekmen, D. C. Son, Y. C. Yang, H. Kim, D. H. Moon, G. Oh, J. Goh, T. J. Kim, S. Cho, S. Choi, Y. Go, D. Gyun, S. Ha, B. Hong, Y. Jo, K. Lee, K. S. Lee, S. Lee, J. Lim, S. K. Park, Y. Roh, H. S. Kim, J. Almond, J. Kim, J. S. Kim, H. Lee, K. Lee, K. Nam, S. B. Oh, B. C. Radburn-Smith, S. h. Seo, U. K. Yang, H. D. Yoo, G. B. Yu, D. Jeon, H. Kim, J. H. Kim, J. S. H. Lee, I. C. Park, Y. Choi, C. Hwang, J. Lee, I. Yu, V. Dudenas, A. Juodagalvis, J. Vaitkus, I. Ahmed, Z. A. Ibrahim, F. Maulida, M. A. B. Md Ali, F. Mohamad Idris, W. A. T. Wan Abdullah, M. N. Yusli, Z. Zolkapli, H. Castilla-Valdez, E. De La Cruz-Burelo, M. C. Duran-Osuna, I. Heredia-De La Cruz, R. Lopez-Fernandez, J. Mejia Guisao, R. I. Rabadan-Trejo, G. Ramirez-Sanchez, R Reyes-Almanza, A. Sanchez-Hernandez, S. Carrillo Moreno, C. Oropeza Barrera, F. Vazquez Valencia, J. Eysermans, I. Pedraza, H. A. Salazar Ibarguen, C. Uribe Estrada, A. Morelos Pineda, D. Krofcheck, S. Bheesette, P. H. Butler, A. Ahmad, M. Ahmad, M. I. Asghar, Q. Hassan, H. R. Hoorani, A. Saddique, M. A. Shah, M. Shoaib, M. Waqas, H. Bialkowska, M. Bluj, B. Boimska, T. Frueboes, M. Górski, M. Kazana, K. Nawrocki, M. Szleper, P. Traczyk, P. Zalewski, K. Bunkowski, A. Byszuk, K. Doroba, A. Kalinowski, M. Konecki, J. Krolikowski, M. Misiura, M. Olszewski, A. Pyskir, M. Walczak, P. Bargassa, C. Beirão Da Cruz E Silva, A. Di Francesco, P. Faccioli, B. Galinhas, M. Gallinaro, J. Hollar, N. Leonardo, L. Lloret Iglesias, M. V. Nemallapudi, J. Seixas, G. Strong, O. Toldaiev, D. Vadruccio, J. Varela, M. Gavrilenko, I. Golutvin, V. Karjavin, I. Kashunin, V. Korenkov, G. Kozlov, A. Lanev, A. Malakhov, V. Matveev, V. V. Mitsyn, P. Moisenz, V. Palichik, V. Perelygin, S. Shmatov, V. Smirnov, V. Trofimov, B. S. Yuldashev, A. Zarubin, V. Zhiltsov, V. Golovtsov, Y. Ivanov, V. Kim, E. Kuznetsova, P. Levchenko, V. Murzin, V. Oreshkin, I. Smirnov, D. Sosnov, V. Sulimov, L. Uvarov, S. Vavilov, A. Vorobyev, Yu. Andreev, A. Dermenev, S. Gninenko, N. Golubev, A. Karneyeu, M. Kirsanov, N. Krasnikov, A. Pashenkov, D. Tlisov, A. Toropin, V. Epshteyn, V. Gavrilov, N. Lychkovskaya, V. Popov, I. Pozdnyakov, G. Safronov, A. Spiridonov, A. Stepennov, V. Stolin, M. Toms, E. Vlasov, A. Zhokin, T. Aushev, R. Chistov, M. Danilov, P. Parygin, D. Philippov, S. Polikarpov, E. Tarkovskii, V. Andreev, M. Azarkin, I. Dremin, M. Kirakosyan, S. V. Rusakov, A. Terkulov, A. Baskakov, A. Belyaev, E. Boos, V. Bunichev, M. Dubinin, L. Dudko, A. Gribushin, V. Klyukhin, N. Korneeva, I. Lokhtin, I. Miagkov, S. Obraztsov, M. Perfilov, V. Savrin, P. Volkov, V. Blinov, T. Dimova, L. Kardapoltsev, D. Shtol, Y. Skovpen, I. Azhgirey, I. Bayshev, S. Bitioukov, D. Elumakhov, A. Godizov, V. Kachanov, A. Kalinin, D. Konstantinov, P. Mandrik, V. Petrov, R. Ryutin, S. Slabospitskii, A. Sobol, S. Troshin, N. Tyurin, A. Uzunian, A. Volkov, A. Babaev, S. Baidali, P. Adzic, P. Cirkovic, D. Devetak, M. Dordevic, J. Milosevic, J. Alcaraz Maestre, A. Álvarez Fernández, I. Bachiller, M. Barrio Luna, J. A. Brochero Cifuentes, M. Cerrada, N. Colino, B. De La Cruz, A. Delgado Peris, C. Fernandez Bedoya, J. P. Fernández Ramos, J. Flix, M. C. Fouz, O. Gonzalez Lopez, S. Goy Lopez, J. M. Hernandez, M. I. Josa, D. Moran, A. Pérez-Calero Yzquierdo, J. Puerta Pelayo, I. Redondo, L. Romero, M. S. Soares, A. Triossi, C. Albajar, J. F. de Trocóniz, J. Cuevas, C. Erice, J. Fernandez Menendez, S. Folgueras, I. Gonzalez Caballero, J. R. González Fernández, E. Palencia Cortezon, V. Rodríguez Bouza, S. Sanchez Cruz, P. Vischia, J. M. Vizan Garcia, I. J. Cabrillo, A. Calderon, B. Chazin Quero, J. Duarte Campderros, M. Fernandez, P. J. Fernández Manteca, A. García Alonso, J. Garcia-Ferrero, G. Gomez, A. Lopez Virto, J. Marco, C. Martinez Rivero, P. Martinez Ruiz del Arbol, F. Matorras, J. Piedra Gomez, C. Prieels, T. Rodrigo, A. Ruiz-Jimeno, L. Scodellaro, N. Trevisani, I. Vila, R. Vilar Cortabitarte, D. Abbaneo, B. Akgun, E. Auffray, P. Baillon, A. H. Ball, D. Barney, J. Bendavid, M. Bianco, A. Bocci, C. Botta, T. Camporesi, M. Cepeda, G. Cerminara, E. Chapon, Y. Chen, G. Cucciati, D. d’Enterria, A. Dabrowski, V. Daponte, A. David, A. De Roeck, N. Deelen, M. Dobson, T. du Pree, M. Dünser, N. Dupont, A. Elliott-Peisert, P. Everaerts, F. Fallavollita, D. Fasanella, G. Franzoni, J. Fulcher, W. Funk, D. Gigi, A. Gilbert, K. Gill, F. Glege, M. Guilbaud, D. Gulhan, J. Hegeman, V. Innocente, A. Jafari, P. Janot, O. Karacheban, J. Kieseler, A. Kornmayer, M. Krammer, C. Lange, P. Lecoq, C. Lourenço, L. Malgeri, M. Mannelli, F. Meijers, J. A. Merlin, S. Mersi, E. Meschi, P. Milenovic, F. Moortgat, M. Mulders, J. Ngadiuba, S. Orfanelli, L. Orsini, F. Pantaleo, L. Pape, E. Perez, M. Peruzzi, A. Petrilli, G. Petrucciani, A. Pfeiffer, M. Pierini, F. M. Pitters, D. Rabady, A. Racz, T. Reis, G. Rolandi, M. Rovere, H. Sakulin, C. Schäfer, C. Schwick, M. Seidel, M. Selvaggi, A. Sharma, P. Silva, P. Sphicas, A. Stakia, J. Steggemann, M. Tosi, D. Treille, A. Tsirou, V. Veckalns, W. D. Zeuner, L. Caminada, K. Deiters, W. Erdmann, R. Horisberger, Q. Ingram, H. C. Kaestli, D. Kotlinski, U. Langenegger, T. Rohe, S. A. Wiederkehr, M. Backhaus, L. Bäni, P. Berger, N. Chernyavskaya, G. Dissertori, M. Dittmar, M. Donegà, C. Dorfer, C. Grab, C. Heidegger, D. Hits, J. Hoss, T. Klijnsma, W. Lustermann, R. A. Manzoni, M. Marionneau, M. T. Meinhard, F. Micheli, P. Musella, F. Nessi-Tedaldi, J. Pata, F. Pauss, G. Perrin, L. Perrozzi, S. Pigazzini, M. Quittnat, D. Ruini, D. A. Sanz Becerra, M. Schönenberger, L. Shchutska, V. R. Tavolaro, K. Theofilatos, M. L. Vesterbacka Olsson, R. Wallny, D. H. Zhu, T. K. Aarrestad, C. Amsler, D. Brzhechko, M. F. Canelli, A. De Cosa, R. Del Burgo, S. Donato, C. Galloni, T. Hreus, B. Kilminster, I. Neutelings, D. Pinna, G. Rauco, P. Robmann, D. Salerno, K. Schweiger, C. Seitz, Y. Takahashi, A. Zucchetta, Y. H. Chang, K. y. Cheng, T. H. Doan, Sh. Jain, R. Khurana, C. M. Kuo, W. Lin, A. Pozdnyakov, S. S. Yu, P. Chang, Y. Chao, K. F. Chen, P. H. Chen, W.-S. Hou, Arun Kumar, Y. y. Li, R.-S. Lu, E. Paganis, A. Psallidas, A. Steen, J. f. Tsai, B. Asavapibhop, N. Srimanobhas, N. Suwonjandee, M. N. Bakirci, A. Bat, F. Boran, S. Damarseckin, Z. S. Demiroglu, F. Dolek, C. Dozen, S. Girgis, G. Gokbulut, Y. Guler, E. Gurpinar, I. Hos, C. Isik, E. E. Kangal, O. Kara, A. Kayis Topaksu, U. Kiminsu, M. Oglakci, G. Onengut, K. Ozdemir, S. Ozturk, D. Sunar Cerci, B. Tali, U. G. Tok, H. Topakli, S. Turkcapar, I. S. Zorbakir, C. Zorbilmez, B. Isildak, G. Karapinar, M. Yalvac, M. Zeyrek, I. O. Atakisi, E. Gülmez, M. Kaya, O. Kaya, S. Tekten, E. A. Yetkin, M. N. Agaras, S. Atay, A. Cakir, K. Cankocak, Y. Komurcu, S. Sen, B. Grynyov, L. Levchuk, F. Ball, L. Beck, J. J. Brooke, D. Burns, E. Clement, D. Cussans, O. Davignon, H. Flacher, J. Goldstein, G. P. Heath, H. F. Heath, L. Kreczko, D. M. Newbold, S. Paramesvaran, B. Penning, T. Sakuma, D. Smith, V. J. Smith, J. Taylor, A. Titterton, K. W. Bell, A. Belyaev, C. Brew, R. M. Brown, D. Cieri, D. J. A. Cockerill, J. A. Coughlan, K. Harder, S. Harper, J. Linacre, E. Olaiya, D. Petyt, C. H. Shepherd-Themistocleous, A. Thea, I. R. Tomalin, T. Williams, W. J. Womersley, G. Auzinger, R. Bainbridge, P. Bloch, J. Borg, S. Breeze, O. Buchmuller, A. Bundock, S. Casasso, D. Colling, L. Corpe, P. Dauncey, G. Davies, M. Della Negra, R. Di Maria, Y. Haddad, G. Hall, G. Iles, T. James, M. Komm, C. Laner, L. Lyons, A.-M. Magnan, S. Malik, A. Martelli, J. Nash, A. Nikitenko, V. Palladino, M. Pesaresi, A. Richards, A. Rose, E. Scott, C. Seez, A. Shtipliyski, T. Strebler, S. Summers, A. Tapper, K. Uchida, T. Virdee, N. Wardle, D. Winterbottom, J. Wright, S. C. Zenz, J. E. Cole, P. R. Hobson, A. Khan, P. Kyberd, C. K. Mackay, A. Morton, I. D. Reid, L. Teodorescu, S. Zahid, K. Call, J. Dittmann, K. Hatakeyama, H. Liu, C. Madrid, B. Mcmaster, N. Pastika, C. Smith, R. Bartek, A. Dominguez, A. Buccilli, S. I. Cooper, C. Henderson, P. Rumerio, C. West, D. Arcaro, T. Bose, D. Gastler, D. Rankin, C. Richardson, J. Rohlf, L. Sulak, D. Zou, G. Benelli, X. Coubez, D. Cutts, M. Hadley, J. Hakala, U. Heintz, J. M. Hogan, K. H. M. Kwok, E. Laird, G. Landsberg, J. Lee, Z. Mao, M. Narain, J. Pazzini, S. Piperov, S. Sagir, R. Syarif, E. Usai, D. Yu, R. Band, C. Brainerd, R. Breedon, D. Burns, M. Calderon De La Barca Sanchez, M. Chertok, J. Conway, R. Conway, P. T. Cox, R. Erbacher, C. Flores, G. Funk, W. Ko, O. Kukral, R. Lander, C. Mclean, M. Mulhearn, D. Pellett, J. Pilot, S. Shalhout, M. Shi, D. Stolp, D. Taylor, K. Tos, M. Tripathi, Z. Wang, M. Bachtis, C. Bravo, R. Cousins, A. Dasgupta, A. Florent, J. Hauser, M. Ignatenko, N. Mccoll, S. Regnard, D. Saltzberg, C. Schnaible, V. Valuev, E. Bouvier, K. Burt, R. Clare, J. W. Gary, S. M. A. Ghiasi Shirazi, G. Hanson, G. Karapostoli, E. Kennedy, F. Lacroix, O. R. Long, M. Olmedo Negrete, M. I. Paneva, W. Si, L. Wang, H. Wei, S. Wimpenny, B. R. Yates, J. G. Branson, S. Cittolin, M. Derdzinski, R. Gerosa, D. Gilbert, B. Hashemi, A. Holzner, D. Klein, G. Kole, V. Krutelyov, J. Letts, M. Masciovecchio, D. Olivito, S. Padhi, M. Pieri, M. Sani, V. Sharma, S. Simon, M. Tadel, A. Vartak, S. Wasserbaech, J. Wood, F. Würthwein, A. Yagil, G. Zevi Della Porta, N. Amin, R. Bhandari, J. Bradmiller-Feld, C. Campagnari, M. Citron, A. Dishaw, V. Dutta, M. Franco Sevilla, L. Gouskos, R. Heller, J. Incandela, A. Ovcharova, H. Qu, J. Richman, D. Stuart, I. Suarez, S. Wang, J. Yoo, D. Anderson, A. Bornheim, J. M. Lawhorn, H. B. Newman, T. Q. Nguyen, M. Spiropulu, J. R. Vlimant, R. Wilkinson, S. Xie, Z. Zhang, R. Y. Zhu, M. B. Andrews, T. Ferguson, T. Mudholkar, M. Paulini, M. Sun, I. Vorobiev, M. Weinberg, J. P. Cumalat, W. T. Ford, F. Jensen, A. Johnson, M. Krohn, S. Leontsinis, E. MacDonald, T. Mulholland, K. Stenson, K. A. Ulmer, S. R. Wagner, J. Alexander, J. Chaves, Y. Cheng, J. Chu, A. Datta, K. Mcdermott, N. Mirman, J. R. Patterson, D. Quach, A. Rinkevicius, A. Ryd, L. Skinnari, L. Soffi, S. M. Tan, Z. Tao, J. Thom, J. Tucker, P. Wittich, M. Zientek, S. Abdullin, M. Albrow, M. Alyari, G. Apollinari, A. Apresyan, A. Apyan, S. Banerjee, L. A. T. Bauerdick, A. Beretvas, J. Berryhill, P. C. Bhat, G. Bolla, K. Burkett, J. N. Butler, A. Canepa, G. B. Cerati, H. W. K. Cheung, F. Chlebana, M. Cremonesi, J. Duarte, V. D. Elvira, J. Freeman, Z. Gecse, E. Gottschalk, L. Gray, D. Green, S. Grünendahl, O. Gutsche, J. Hanlon, R. M. Harris, S. Hasegawa, J. Hirschauer, Z. Hu, B. Jayatilaka, S. Jindariani, M. Johnson, U. Joshi, B. Klima, M. J. Kortelainen, B. Kreis, S. Lammel, D. Lincoln, R. Lipton, M. Liu, T. Liu, J. Lykken, K. Maeshima, J. M. Marraffino, D. Mason, P. McBride, P. Merkel, S. Mrenna, S. Nahn, V. O’Dell, K. Pedro, O. Prokofyev, G. Rakness, L. Ristori, A. Savoy-Navarro, B. Schneider, E. Sexton-Kennedy, A. Soha, W. J. Spalding, L. Spiegel, S. Stoynev, J. Strait, N. Strobbe, L. Taylor, S. Tkaczyk, N. V. Tran, L. Uplegger, E. W. Vaandering, C. Vernieri, M. Verzocchi, R. Vidal, M. Wang, H. A. Weber, A. Whitbeck, D. Acosta, P. Avery, P. Bortignon, D. Bourilkov, A. Brinkerhoff, L. Cadamuro, A. Carnes, M. Carver, D. Curry, R. D. Field, S. V. Gleyzer, B. M. Joshi, J. Konigsberg, A. Korytov, P. Ma, K. Matchev, H. Mei, G. Mitselmakher, K. Shi, D. Sperka, J. Wang, S. Wang, Y. R. Joshi, S. Linn, A. Ackert, T. Adams, A. Askew, S. Hagopian, V. Hagopian, K. F. Johnson, T. Kolberg, G. Martinez, T. Perry, H. Prosper, A. Saha, A. Santra, V. Sharma, R. Yohay, M. M. Baarmand, V. Bhopatkar, S. Colafranceschi, M. Hohlmann, D. Noonan, M. Rahmani, T. Roy, F. Yumiceva, M. R. Adams, L. Apanasevich, D. Berry, R. R. Betts, R. Cavanaugh, X. Chen, S. Dittmer, O. Evdokimov, C. E. Gerber, D. A. Hangal, D. J. Hofman, K. Jung, J. Kamin, C. Mills, I. D. Sandoval Gonzalez, M. B. Tonjes, N. Varelas, H. Wang, X. Wang, Z. Wu, J. Zhang, M. Alhusseini, B. Bilki, W. Clarida, K. Dilsiz, S. Durgut, R. P. Gandrajula, M. Haytmyradov, V. Khristenko, J.-P. Merlo, A. Mestvirishvili, A. Moeller, J. Nachtman, H. Ogul, Y. Onel, F. Ozok, A. Penzo, C. Snyder, E. Tiras, J. Wetzel, B. Blumenfeld, A. Cocoros, N. Eminizer, D. Fehling, L. Feng, A. V. Gritsan, W. T. Hung, P. Maksimovic, J. Roskes, U. Sarica, M. Swartz, M. Xiao, C. You, A. Al-bataineh, P. Baringer, A. Bean, S. Boren, J. Bowen, A. Bylinkin, J. Castle, S. Khalil, A. Kropivnitskaya, D. Majumder, W. Mcbrayer, M. Murray, C. Rogan, S. Sanders, E. Schmitz, J. D. Tapia Takaki, Q. Wang, A. Ivanov, K. Kaadze, D. Kim, Y. Maravin, D. R. Mendis, T. Mitchell, A. Modak, A. Mohammadi, L. K. Saini, N. Skhirtladze, F. Rebassoo, D. Wright, A. Baden, O. Baron, A. Belloni, S. C. Eno, Y. Feng, C. Ferraioli, N. J. Hadley, S. Jabeen, G. Y. Jeng, R. G. Kellogg, J. Kunkle, A. C. Mignerey, F. Ricci-Tam, Y. H. Shin, A. Skuja, S. C. Tonwar, K. Wong, D. Abercrombie, B. Allen, V. Azzolini, A. Baty, G. Bauer, R. Bi, S. Brandt, W. Busza, I. A. Cali, M. D’Alfonso, Z. Demiragli, G. Gomez Ceballos, M. Goncharov, P. Harris, D. Hsu, M. Hu, Y. Iiyama, G. M. Innocenti, M. Klute, D. Kovalskyi, Y.-J. Lee, P. D. Luckey, B. Maier, A. C. Marini, C. Mcginn, C. Mironov, S. Narayanan, X. Niu, C. Paus, C. Roland, G. Roland, G. S. F. Stephans, K. Sumorok, K. Tatar, D. Velicanu, J. Wang, T. W. Wang, B. Wyslouch, S. Zhaozhong, A. C. Benvenuti, R. M. Chatterjee, A. Evans, P. Hansen, S. Kalafut, Y. Kubota, Z. Lesko, J. Mans, S. Nourbakhsh, N. Ruckstuhl, R. Rusack, J. Turkewitz, M. A. Wadud, J. G. Acosta, S. Oliveros, E. Avdeeva, K. Bloom, D. R. Claes, C. Fangmeier, F. Golf, R. Gonzalez Suarez, R. Kamalieddin, I. Kravchenko, J. Monroy, J. E. Siado, G. R. Snow, B. Stieger, A. Godshalk, C. Harrington, I. Iashvili, A. Kharchilava, D. Nguyen, A. Parker, S. Rappoccio, B. Roozbahani, E. Barberis, C. Freer, A. Hortiangtham, D. M. Morse, T. Orimoto, R. Teixeira De Lima, T. Wamorkar, B. Wang, A. Wisecarver, D. Wood, S. Bhattacharya, O. Charaf, K. A. Hahn, N. Mucia, N. Odell, M. H. Schmitt, K. Sung, M. Trovato, M. Velasco, R. Bucci, N. Dev, M. Hildreth, K. Hurtado Anampa, C. Jessop, D. J. Karmgard, N. Kellams, K. Lannon, W. Li, N. Loukas, N. Marinelli, F. Meng, C. Mueller, Y. Musienko, M. Planer, A. Reinsvold, R. Ruchti, P. Siddireddy, G. Smith, S. Taroni, M. Wayne, A. Wightman, M. Wolf, A. Woodard, J. Alimena, L. Antonelli, B. Bylsma, L. S. Durkin, S. Flowers, B. Francis, A. Hart, C. Hill, W. Ji, T. Y. Ling, W. Luo, B. L. Winer, H. W. Wulsin, S. Cooperstein, P. Elmer, J. Hardenbrook, P. Hebda, S. Higginbotham, A. Kalogeropoulos, D. Lange, M. T. Lucchini, J. Luo, D. Marlow, K. Mei, I. Ojalvo, J. Olsen, C. Palmer, P. Piroué, J. Salfeld-Nebgen, D. Stickland, C. Tully, S. Malik, S. Norberg, A. Barker, V. E. Barnes, L. Gutay, M. Jones, A. W. Jung, A. Khatiwada, B. Mahakud, D. H. Miller, N. Neumeister, C. C. Peng, H. Qiu, J. F. Schulte, J. Sun, F. Wang, R. Xiao, W. Xie, T. Cheng, J. Dolen, N. Parashar, Z. Chen, K. M. Ecklund, S. Freed, F. J. M. Geurts, M. Kilpatrick, W. Li, B. Michlin, B. P. Padley, J. Roberts, J. Rorie, W. Shi, Z. Tu, J. Zabel, A. Zhang, A. Bodek, P. de Barbaro, R. Demina, Y. t. Duh, J. L. Dulemba, C. Fallon, T. Ferbel, M. Galanti, A. Garcia-Bellido, J. Han, O. Hindrichs, A. Khukhunaishvili, K. H. Lo, P. Tan, R. Taus, M. Verzetti, A. Agapitos, J. P. Chou, Y. Gershtein, T. A. Gómez Espinosa, E. Halkiadakis, M. Heindl, E. Hughes, S. Kaplan, R. Kunnawalkam Elayavalli, S. Kyriacou, A. Lath, R. Montalvo, K. Nash, M. Osherson, H. Saka, S. Salur, S. Schnetzer, D. Sheffield, S. Somalwar, R. Stone, S. Thomas, P. Thomassen, M. Walker, A. G. Delannoy, J. Heideman, G. Riley, K. Rose, S. Spanier, K. Thapa, O. Bouhali, A. Castaneda Hernandez, A. Celik, M. Dalchenko, M. De Mattia, A. Delgado, S. Dildick, R. Eusebi, J. Gilmore, T. Huang, T. Kamon, S. Luo, R. Mueller, Y. Pakhotin, R. Patel, A. Perloff, L. Perniè, D. Rathjens, A. Safonov, A. Tatarinov, N. Akchurin, J. Damgov, F. De Guio, P. R. Dudero, S. Kunori, K. Lamichhane, S. W. Lee, T. Mengke, S. Muthumuni, T. Peltola, S. Undleeb, I. Volobouev, Z. Wang, S. Greene, A. Gurrola, R. Janjam, W. Johns, C. Maguire, A. Melo, H. Ni, K. Padeken, J. D. Ruiz Alvarez, P. Sheldon, S. Tuo, J. Velkovska, M. Verweij, Q. Xu, M. W. Arenton, P. Barria, B. Cox, R. Hirosky, M. Joyce, A. Ledovskoy, H. Li, C. Neu, T. Sinthuprasith, Y. Wang, E. Wolfe, F. Xia, R. Harr, P. E. Karchin, N. Poudyal, J. Sturdy, P. Thapa, S. Zaleski, M. Brodski, J. Buchanan, C. Caillol, D. Carlsmith, S. Dasu, L. Dodd, S. Duric, B. Gomber, M. Grothe, M. Herndon, A. Hervé, U. Hussain, P. Klabbers, A. Lanaro, A. Levine, K. Long, R. Loveless, T. Ruggles, A. Savin, N. Smith, W. H. Smith, N. Woods, CMS Collaboration

**Affiliations:** 1 Yerevan Physics Institute, Yerevan, Armenia; 2 Institut für Hochenergiephysik, Vienna, Austria; 3 Institute for Nuclear Problems, Minsk, Belarus; 4 Universiteit Antwerpen, Antwerp, Belgium; 5 Vrije Universiteit Brussel, Brussels, Belgium; 6 Université Libre de Bruxelles, Brussels, Belgium; 7 Ghent University, Ghent, Belgium; 8 Université Catholique de Louvain, Louvain-la-Neuve, Belgium; 9 Centro Brasileiro de Pesquisas Fisicas, Rio de Janeiro, Brazil; 10 Universidade do Estado do Rio de Janeiro, Rio de Janeiro, Brazil; 11 Universidade Estadual Paulista, Universidade Federal do ABC, São Paulo, Brazil; 12 Institute for Nuclear Research and Nuclear Energy, Bulgarian Academy of Sciences, Sofia, Bulgaria; 13 University of Sofia, Sofia, Bulgaria; 14 Beihang University, Beijing, China; 15 Institute of High Energy Physics, Beijing, China; 16 State Key Laboratory of Nuclear Physics and Technology, Peking University, Beijing, China; 17 Tsinghua University, Beijing, China; 18 Universidad de Los Andes, Bogotá, Colombia; 19 University of Split, Faculty of Electrical Engineering, Mechanical Engineering and Naval Architecture, Split, Croatia; 20 University of Split, Faculty of Science, Split, Croatia; 21 Institute Rudjer Boskovic, Zagreb, Croatia; 22 University of Cyprus, Nicosia, Cyprus; 23 Charles University, Prague, Czech Republic; 24 Escuela Politecnica Nacional, Quito, Ecuador; 25 Universidad San Francisco de Quito, Quito, Ecuador; 26 Academy of Scientific Research and Technology of the Arab Republic of Egypt, Egyptian Network of High Energy Physics, Cairo, Egypt; 27 National Institute of Chemical Physics and Biophysics, Tallinn, Estonia; 28 Department of Physics, University of Helsinki, Helsinki, Finland; 29 Helsinki Institute of Physics, Helsinki, Finland; 30 Lappeenranta University of Technology, Lappeenranta, Finland; 31 IRFU, CEA, Université Paris-Saclay, Gif-sur-Yvette, France; 32 Laboratoire Leprince-Ringuet, Ecole polytechnique, CNRS/IN2P3, Université Paris-Saclay, Palaiseau, France; 33 Université de Strasbourg, CNRS, IPHC UMR 7178, Strasbourg, France; 34 Centre de Calcul de l’Institut National de Physique Nucleaire et de Physique des Particules, CNRS/IN2P3, Villeurbanne, France; 35 Université de Lyon, Université Claude Bernard Lyon 1, CNRS-IN2P3, Institut de Physique Nucléaire de Lyon, Villeurbanne, France; 36 Georgian Technical University, Tbilisi, Georgia; 37 Tbilisi State University, Tbilisi, Georgia; 38 RWTH Aachen University, I. Physikalisches Institut, Aachen, Germany; 39 RWTH Aachen University, III. Physikalisches Institut A, Aachen, Germany; 40 RWTH Aachen University, III. Physikalisches Institut B, Aachen, Germany; 41 Deutsches Elektronen-Synchrotron, Hamburg, Germany; 42 University of Hamburg, Hamburg, Germany; 43 Karlsruher Institut für Technology, Karlsruhe, Germany; 44 Institute of Nuclear and Particle Physics (INPP), NCSR Demokritos, Aghia Paraskevi, Greece; 45 National and Kapodistrian University of Athens, Athens, Greece; 46 National Technical University of Athens, Athens, Greece; 47 University of Ioánnina, Ioánnina, Greece; 48 MTA-ELTE Lendület CMS Particle and Nuclear Physics Group, Eötvös Loránd University, Budapest, Hungary; 49 Wigner Research Centre for Physics, Budapest, Hungary; 50 Institute of Nuclear Research ATOMKI, Debrecen, Hungary; 51 Institute of Physics, University of Debrecen, Debrecen, Hungary; 52 Indian Institute of Science (IISc), Bangalore, India; 53 National Institute of Science Education and Research, HBNI, Bhubaneswar, India; 54 Panjab University, Chandigarh, India; 55 University of Delhi, Delhi, India; 56 Saha Institute of Nuclear Physics, HBNI, Kolkata, India; 57 Indian Institute of Technology Madras, Madras, India; 58 Bhabha Atomic Research Centre, Mumbai, India; 59 Tata Institute of Fundamental Research-A, Mumbai, India; 60 Tata Institute of Fundamental Research-B, Mumbai, India; 61 Indian Institute of Science Education and Research (IISER), Pune, India; 62 Institute for Research in Fundamental Sciences (IPM), Tehran, Iran; 63 University College Dublin, Dublin, Ireland; 64 INFN Sezione di Bari, Università di Bari, Politecnico di Bari, Bari, Italy; 65 INFN Sezione di Bologna, Università di Bologna, Bologna, Italy; 66 INFN Sezione di Catania, Università di Catania, Catania, Italy; 67 INFN Sezione di Firenze, Università di Firenze, Florence, Italy; 68 INFN Laboratori Nazionali di Frascati, Frascati, Italy; 69 INFN Sezione di Genova, Università di Genova, Genoa, Italy; 70 INFN Sezione di Milano-Bicocca, Università di Milano-Bicocca, Milan, Italy; 71 INFN Sezione di Napoli, Università di Napoli ‘Federico II’, Naples, Italy, Università della Basilicata, Potenza, Italy, Università G. Marconi, Rome, Italy; 72 INFN Sezione di Padova, Università di Padova, Padua, Italy, Università di Trento, Trento, Italy; 73 INFN Sezione di Pavia, Università di Pavia, Pavia, Italy; 74 INFN Sezione di Perugia, Università di Perugia, Perugia, Italy; 75 INFN Sezione di Pisa, Università di Pisa, Scuola Normale Superiore di Pisa, Pisa, Italy; 76 INFN Sezione di Roma, Sapienza Università di Roma, Rome, Italy; 77 INFN Sezione di Torino, Università di Torino, Torino, Italy, Università del Piemonte Orientale, Novara, Italy; 78 INFN Sezione di Trieste, Università di Trieste, Trieste, Italy; 79 Kyungpook National University, Daegu, South Korea; 80 Institute for Universe and Elementary Particles, Chonnam National University, Kwangju, Korea; 81 Hanyang University, Seoul, Korea; 82 Korea University, Seoul, Korea; 83 Sejong University, Seoul, Korea; 84 Seoul National University, Seoul, Korea; 85 University of Seoul, Seoul, Korea; 86 Sungkyunkwan University, Suwon, Korea; 87 Vilnius University, Vilnius, Lithuania; 88 National Centre for Particle Physics, Universiti Malaya, Kuala Lumpur, Malaysia; 89 Centro de Investigacion y de Estudios Avanzados del IPN, Mexico City, Mexico; 90 Universidad Iberoamericana, Mexico City, Mexico; 91 Benemerita Universidad Autonoma de Puebla, Puebla, Mexico; 92 Universidad Autónoma de San Luis Potosí, San Luis Potosí, Mexico; 93 University of Auckland, Auckland, New Zealand; 94 University of Canterbury, Christchurch, New Zealand; 95 National Centre for Physics, Quaid-I-Azam University, Islamabad, Pakistan; 96 National Centre for Nuclear Research, Swierk, Poland; 97 Institute of Experimental Physics, Faculty of Physics, University of Warsaw, Warsaw, Poland; 98 Laboratório de Instrumentação e Física Experimental de Partículas, Lisbon, Portugal; 99 Joint Institute for Nuclear Research, Dubna, Russia; 100 Petersburg Nuclear Physics Institute, Gatchina (St. Petersburg), Russia; 101 Institute for Nuclear Research, Moscow, Russia; 102 Institute for Theoretical and Experimental Physics, Moscow, Russia; 103 Moscow Institute of Physics and Technology, Moscow, Russia; 104 National Research Nuclear University ‘Moscow Engineering Physics Institute’ (MEPhI), Moscow, Russia; 105 P.N. Lebedev Physical Institute, Moscow, Russia; 106 Skobeltsyn Institute of Nuclear Physics, Lomonosov Moscow State University, Moscow, Russia; 107 Novosibirsk State University (NSU), Novosibirsk, Russia; 108 State Research Center of Russian Federation, Institute for High Energy Physics of NRC “Kurchatov Institute”, Protvino, Russia; 109 National Research Tomsk Polytechnic University, Tomsk, Russia; 110 Faculty of Physics and Vinca Institute of Nuclear Sciences, University of Belgrade, Belgrade, Serbia; 111 Centro de Investigaciones Energéticas Medioambientales y Tecnológicas (CIEMAT), Madrid, Spain; 112 Universidad Autónoma de Madrid, Madrid, Spain; 113 Universidad de Oviedo, Oviedo, Spain; 114 Instituto de Física de Cantabria (IFCA), CSIC-Universidad de Cantabria, Santander, Spain; 115 CERN, European Organization for Nuclear Research, Geneva, Switzerland; 116 Paul Scherrer Institut, Villigen, Switzerland; 117 ETH Zurich-Institute for Particle Physics and Astrophysics (IPA), Zurich, Switzerland; 118 Universität Zürich, Zurich, Switzerland; 119 National Central University, Chung-Li, Taiwan; 120 National Taiwan University (NTU), Taipei, Taiwan; 121 Faculty of Science, Department of Physics, Chulalongkorn University, Bangkok, Thailand; 122 Physics Department, Science and Art Faculty, Çukurova University, Adana, Turkey; 123 Middle East Technical University, Physics Department, Ankara, Turkey; 124 Bogazici University, Istanbul, Turkey; 125 Istanbul Technical University, Istanbul, Turkey; 126 Institute for Scintillation Materials of National Academy of Science of Ukraine, Kharkov, Ukraine; 127 National Scientific Center, Kharkov Institute of Physics and Technology, Kharkov, Ukraine; 128 University of Bristol, Bristol, UK; 129 Rutherford Appleton Laboratory, Didcot, UK; 130 Imperial College, London, UK; 131 Brunel University, Uxbridge, UK; 132 Baylor University, Waco, USA; 133 Catholic University of America, Washington, DC, USA; 134 The University of Alabama, Tuscaloosa, USA; 135 Boston University, Boston, USA; 136 Brown University, Providence, USA; 137 University of California, Davis, Davis, USA; 138 University of California, Los Angeles, USA; 139 University of California, Riverside, Riverside, USA; 140 University of California, San Diego, La Jolla, USA; 141 Department of Physics, University of California, Santa Barbara, Santa Barbara, USA; 142 California Institute of Technology, Pasadena, USA; 143 Carnegie Mellon University, Pittsburgh, USA; 144 University of Colorado Boulder, Boulder, USA; 145 Cornell University, Ithaca, USA; 146 Fermi National Accelerator Laboratory, Batavia, USA; 147 University of Florida, Gainesville, USA; 148 Florida International University, Miami, USA; 149 Florida State University, Tallahassee, USA; 150 Florida Institute of Technology, Melbourne, USA; 151 University of Illinois at Chicago (UIC), Chicago, USA; 152 The University of Iowa, Iowa City, USA; 153 Johns Hopkins University, Baltimore, USA; 154 The University of Kansas, Lawrence, USA; 155 Kansas State University, Manhattan, USA; 156 Lawrence Livermore National Laboratory, Livermore, USA; 157 University of Maryland, College Park, USA; 158 Massachusetts Institute of Technology, Cambridge, USA; 159 University of Minnesota, Minneapolis, USA; 160 University of Mississippi, Oxford, USA; 161 University of Nebraska-Lincoln, Lincoln, USA; 162 State University of New York at Buffalo, Buffalo, USA; 163 Northeastern University, Boston, USA; 164 Northwestern University, Evanston, USA; 165 University of Notre Dame, Notre Dame, USA; 166 The Ohio State University, Columbus, USA; 167 Princeton University, Princeton, USA; 168 University of Puerto Rico, Mayagüez, USA; 169 Purdue University, West Lafayette, USA; 170 Purdue University Northwest, Hammond, USA; 171 Rice University, Houston, USA; 172 University of Rochester, Rochester, USA; 173 Rutgers, The State University of New Jersey, Piscataway, USA; 174 University of Tennessee, Knoxville, USA; 175 Texas A&M University, College Station, USA; 176 Texas Tech University, Lubbock, USA; 177 Vanderbilt University, Nashville, USA; 178 University of Virginia, Charlottesville, USA; 179 Wayne State University, Detroit, USA; 180 University of Wisconsin-Madison, Madison, WI USA; 181 CERN, 1211 Geneva 23, Switzerland

## Abstract

Measurements of normalized differential cross sections as functions of the multiplicity and kinematic variables of charged-particle tracks from the underlying event in top quark and antiquark pair production are presented. The measurements are performed in proton-proton collisions at a center-of-mass energy of 13$~\text {Te}\text {V}$, and are based on data collected by the CMS experiment at the LHC in 2016 corresponding to an integrated luminosity of 35.9$~\text {fb}^{-1}$. Events containing one electron, one muon, and two jets from the hadronization and fragmentation of $\mathrm {b}$ quarks are used. These measurements characterize, for the first time, properties of the underlying event in top quark pair production and show no deviation from the universality hypothesis at energy scales typically above twice the top quark mass.

## Introduction

At the LHC, top quark and antiquark pairs (${\mathrm {t}\overline{\mathrm {t}}}$) are dominantly produced in the scattering of the proton constituents via quantum chromodynamics (QCD) at an energy scale (*Q*) of about two times the $\mathrm {t}$ quark mass ($m_\mathrm {t}$). The properties of the $\mathrm {t}$ quark can be studied directly from its decay products, as it decays before hadronizing. Mediated by the electroweak interaction, the $\mathrm {t}$ quark decay yields a $\mathrm {W}$ boson and a quark, the latter carrying the QCD color charge of the mother particle. Given the large branching fraction for the decay into a bottom quark, $\mathcal {B}(\mathrm {t}\rightarrow \mathrm {W}\mathrm {b})=0.957\pm 0.034$ [1], in this analysis we assume that each $\mathrm {t}$ or $\overline{\mathrm {t}}$ quark yields a corresponding bottom ($\mathrm {b}$) or antibottom ($\overline{\mathrm {b}}$) quark in its decay. Other quarks may also be produced, in a color-singlet state, if a $\mathrm {W}\rightarrow \mathrm {q} \overline{\mathrm {q}} ^\prime$ decay occurs. Being colored, these quarks will fragment and hadronize giving rise to an experimental signature with jets. Thus, when performing precision measurements of $\mathrm {t}$ quark properties at hadron colliders, an accurate description of the fragmentation and hadronization of the quarks from the hard scatter process as well as of the “underlying event” (UE), defined below, is essential. First studies of the fragmentation and hadronization of the $\mathrm {b}$ quarks in ${\mathrm {t}\overline{\mathrm {t}}}$ events have been reported in Refs. [2, 3]. In this paper, we present the first measurement of the properties of the UE in ${\mathrm {t}\overline{\mathrm {t}}}$ events at a scale $Q\ge 2m_\mathrm {t}$.

The UE is defined as any hadronic activity that cannot be attributed to the particles stemming from the hard scatter, and in this case from ${\mathrm {t}\overline{\mathrm {t}}}$ decays. Because of energy-momentum conservation, the UE constitutes the recoil against the ${\mathrm {t}\overline{\mathrm {t}}}$ system. In this study, the hadronization products of initial- and final-state radiation (ISR and FSR) that cannot be associated to the particles from the ${\mathrm {t}\overline{\mathrm {t}}}$ decays are probed as part of the UE, even if they can be partially modeled by perturbative QCD. The main contribution to the UE comes from the color exchanges between the beam particles and is modeled in terms of multiparton interactions (MPI), color reconnection (CR), and beam-beam remnants (BBR), whose model parameters can be tuned to minimum bias and Drell–Yan (DY) data.

The study of the UE in ${\mathrm {t}\overline{\mathrm {t}}}$ events provides a direct test of its universality at higher energy scales than those probed in minimum bias or DY events. This is relevant as a direct probe of CR, which is needed to confine the initial QCD color charge of the $\mathrm {t}$ quark into color-neutral states. The CR mainly occurs between one of the products of the fragmentation of the $\mathrm {b}$ quark from the $\mathrm {t}$ quark decay and the proton remnants. This is expected to induce an ambiguity in the origin of some of the final states present in a bottom quark jet [4–6]. The impact of these ambiguities in the measurement of $\mathrm {t}$ quark properties is evaluated through phenomenological models that need to be tuned to the data. Recent examples of the impact that different model parameters have on $m_\mathrm {t}$ can be found in Refs. [7, 8].

The analysis is performed using final states where both of the $\mathrm {W}$ bosons decay to leptons, yielding one electron and one muon with opposite charge sign, and the corresponding neutrinos. In addition, two $\mathrm {b}$ jets are required in the selection, as expected from the ${\mathrm {t}\overline{\mathrm {t}}} \rightarrow (\mathrm {e}\nu \mathrm {b})(\mu \nu \mathrm {b})$ decay. This final state is chosen because of its expected high purity and because the products of the hard process can be distinguished with high efficiency and small contamination from objects not associated with $\mathrm {t}$ quark decays, e.g., jets from ISR.

After discussing the experimental setup in Sect. 2, and the signal and background modeling in Sect. 3, we present the strategy employed to select the events in Sect. 4 and to measure the UE contribution in each selected event in Sect. 5. The measurements are corrected to a particle-level definition using the method described in Sect. 6 and the associated systematic uncertainties are discussed in Sect. 7. Finally, in Sect. 8, the results are discussed and compared to predictions from different Monte Carlo (MC) simulations. The measurements are summarized in Sect. 9.

## The CMS detector

The central feature of the CMS apparatus is a superconducting solenoid of 6$~\text {m}$ internal diameter, providing a magnetic field of 3.8$~\text {T}$ parallel to the beam direction.

Within the solenoid volume are a silicon pixel and strip tracker, a lead tungstate crystal electromagnetic calorimeter (ECAL), and a brass and scintillator hadron calorimeter (HCAL), each composed of a barrel and two endcap sections. A preshower detector, consisting of two planes of silicon sensors interleaved with about three radiation lengths of lead, is located in front of the endcap regions of the ECAL. Hadron forward calorimeters, using steel as an absorber and quartz fibers as the sensitive material, extend the pseudorapidity coverage provided by the barrel and endcap detectors from $|\eta | = 3.0$ to 5.2. Muons are detected in the window $|\eta |<2.4$ in gas-ionization detectors embedded in the steel flux-return yoke outside the solenoid.

Charged-particle trajectories with $|\eta |<2.5$ are measured by the tracker system. The particle-flow algorithm [9] is used to reconstruct and identify individual particles in an event, with an optimized combination of information from the various elements of the CMS detector. The energy of the photons is directly obtained from the ECAL measurement, corrected for zero-suppression effects. The energy of the electrons is determined from a combination of the electron momentum at the primary interaction vertex as determined by the tracker, the energy of the corresponding ECAL cluster, and the energy sum of all bremsstrahlung photons spatially compatible with originating from the electron track. The energy of the muons is obtained from the curvature of the corresponding track. The energy of charged hadrons is determined from a combination of their momentum measured in the tracker and the matching ECAL and HCAL energy deposits, corrected for zero-suppression effects and for the response function of the calorimeters to hadronic showers. Finally, the energy of neutral hadrons is obtained from the corresponding corrected ECAL and HCAL energies.

Events of interest are selected using a two-tiered trigger system [10]. The first level, composed of custom hardware processors, uses information from the calorimeters and muon detectors to select events at a rate of around 100$~\text {kHz}$ within a time interval of less than 4$~\upmu \text {s}$. The second level, known as the high-level trigger, consists of a farm of processors running a version of the full event reconstruction software optimized for fast processing, and reduces the event rate to around 1$~\text {kHz}$ before data storage.

A more detailed description of the CMS detector, together with a definition of the coordinate system used and the relevant kinematic variables, can be found in Ref. [11].

## Signal and background modeling

This analysis is based on proton-proton ($\mathrm {p}$ $\mathrm {p}$) collision data at a center-of-mass energy $\sqrt{s}=13~\text {Te}\text {V}$, collected by the CMS detector in 2016 and corresponds to an integrated luminosity of $35.9{~\text {fb}^{-1}}$ [12].

The ${\mathrm {t}\overline{\mathrm {t}}}$ process is simulated with the powheg (v2) generator in the heavy quark production (hvq) mode [13–15]. The NNPDF3.0 next-to-leading-order (NLO) parton distribution functions (PDFs) with the strong coupling parameter $\alpha _S =0.118$ at the $\mathrm {Z}$ boson mass scale ($M_\mathrm {Z}$) [16] are utilized in the matrix-element (ME) calculation. The renormalization and factorization scales, $\mu _\mathrm{R}$ and $\mu _\mathrm{F}$, are set to $m_{\mathrm{T}} =\sqrt{\smash [b]{m_\mathrm {t}^2+p_{\mathrm{T}} ^2}}$, where $m_\mathrm {t}=172.5~\text {Ge}\text {V}$ and $p_{\mathrm{T}}$ is the transverse momentum in the ${\mathrm {t}\overline{\mathrm {t}}}$ rest frame. Parton showering is simulated using pythia8 (v8.219) [17] and the CUETP8M2T4 UE tune [18]. The CUETP8M2T4 tune is based on the CUETP8M1 tune [19] but uses a lower value of $\alpha _S ^{\mathrm{ISR}}(M_\mathrm {Z})=0.1108$ in the parton shower (PS); this value leads to a better description of jet multiplicities in ${\mathrm {t}\overline{\mathrm {t}}}$ events at $\sqrt{s}=8~\text {Te}\text {V}$ [20]. The leading-order (LO) version of the same NNPDF3.0 is used in the PS and MPI simulation in the CUETP8M2T4 tune. The cross section used for the ${\mathrm {t}\overline{\mathrm {t}}}$ simulation is $832^{+20}_{-29}~(\text {scale})\pm 35(\text {PDF}+\alpha _S)~\text {pb}$, computed at the next-to-next-to-leading-order (NNLO) plus next-to-next-to-leading-logarithmic accuracy  [21].

Throughout this paper, data are compared to the predictions of different generator settings for the ${\mathrm {t}\overline{\mathrm {t}}}$ process. Table 1 summarizes the main characteristics of the setups and abbreviations used in the paper. Among other UE properties, CR and MPI are modeled differently in the alternative setups considered, hence the interest in comparing them to the data. Three different signal ME generators are used: powheg, MadGraph 5_amc@nlo (v2.2.2) with the FxFx merging scheme [22, 23] for jets from the ME calculations and PS, and sherpa (v2.2.4) [24]. The latter is used in combination with OpenLoops (v1.3.1) [25], and with the CS parton shower based on the Catani–Seymour dipole subtraction scheme [26]. In addition, two different herwig PS versions are used and interfaced with powheg: herwig++  [27] with the EE5C UE tune [28] and the CTEQ6 (L1) [29] PDF set, and herwig 7 [27, 30] with its default tune and the MMHT2014 (LO) [31] PDF set.

**Table 1:** MC simulation settings used for the comparisons with the differential cross section measurements of the UE. The table lists the main characteristics and values used for the most relevant parameters of the generators. The row labeled “Setup designation” shows the definitions of the abbreviations used throughout this paper

Event generator	powheg (v2)	MadGraph 5_amc@nlo (v2.2.2)	sherpa (v2.2.4)
*Matrix element characteristics*			
Mode	hvq	FxFx Merging	OpenLoops
Scales ($\mu _\mathrm{R},\mu _\mathrm{F}$)	$m_{\mathrm{T}}$	$\sum _{\mathrm {t},\overline{\mathrm {t}}}m_{\mathrm{T}}/2$	$\hbox {METS} + \hbox {QCD}$
$\alpha _S (M_\mathrm {Z})$	0.118	0.118	0.118
PDF	NNPDF3.0 NLO	NNPDF3.0 NLO	NNPDF3.0 NNLO
Accuracy	${\mathrm {t}\overline{\mathrm {t}}}$ [NLO]	${\mathrm {t}\overline{\mathrm {t}}} + 0,1,2~\hbox {jets}$ [NLO]	${\mathrm {t}\overline{\mathrm {t}}}$ [NLO]
	1 jet [LO]	3 jets [LO]	
*Parton shower*			
Setup designation	Pw+Py8	MG5_aMC	sherpa
PS	pythia (v8.219)		CS
Tune	CUETP8M2T4		default
PDF	NNPDF2.3 LO		NNPDF3.0 NNLO
($\alpha _S ^{\mathrm{ISR}}(M_\mathrm {Z}),\alpha _S ^{\mathrm{FSR}}(M_\mathrm {Z})$)	(0.1108, 0.1365)		(0.118, 0.118)
ME corrections	on		—
Setup designation	Pw+Hw++	Pw+Hw7	
PS	herwig++	herwig 7	
Tune	EE5C	Default	
PDF	CTEQ6 (L1)	MMHT2014 LO	
($\alpha _S ^{\mathrm{ISR}}(M_\mathrm {Z}),\alpha _S ^{\mathrm{FSR}}(M_\mathrm {Z})$)	(0.1262, 0.1262)	(0.1262, 0.1262)	
ME corrections	Off	On	

Additional variations of the Pw+Py8 sample are used to illustrate the sensitivity of the measurements to different parameters of the UE model. A supplementary table, presented in the appendix, details the parameters that have been changed with respect to the CUETP8M2T4 tune in these additional variations. The variations include extreme models that highlight separately the contributions of MPI and CR to the UE, fine-grained variations of different CR models [5, 32], an alternative MPI model based on the “Rope hadronization” framework describing Lund color strings overlapping in the same area [33, 34], variations of the choice of $\alpha _S (M_\mathrm {Z})$ in the parton shower, and a variation of the values that constitute the CUETP8M2T4 tune, according to their uncertainties.

Background processes are simulated with several generators. The $\mathrm {W}$ $\mathrm {Z}$, $\mathrm {W}$+jets, and $\mathrm {Z}\mathrm {Z}\rightarrow 2\ell 2q$ (where $\ell$ denotes any of the charged leptons e/$\mu$/$\tau$) processes are simulated at NLO, using MadGraph 5_amc@nlo with the FxFx merging. Drell–Yan production, with dilepton invariant mass, $m(\ell \ell )$, greater than 50$~\text {Ge}\text {V}$, is simulated at LO with MadGraph 5_amc@nlo using the so-called MLM matching scheme [35] for jet merging. The powheg (v2) program is furthermore used to simulate the $\mathrm {W}$ $\mathrm {W}$, and $\mathrm {Z}\mathrm {Z}\rightarrow 2\ell 2\nu$ processes [36, 37], while powheg (v1) is used to simulate the $\mathrm {t}$ $\mathrm {W}$ process [38]. The single top quark *t*-channel background is simulated at NLO using powheg (v2) and MadSpin contained in MadGraph 5_amc@nlo (v2.2.2) [39, 40]. The residual $\mathrm {t}$ $\overline{\mathrm {t}}$+V backgrounds, where $\mathrm {V}=\mathrm {W}{}$ or $\mathrm {Z}$, are generated at NLO using MadGraph 5_amc@nlo. The cross sections of the DY and $\mathrm {W}$+jets processes are normalized to the NNLO prediction, computed using fewz (v3.1.b2) [41], and single top quark processes are normalized to the approximate NNLO prediction [42]. Processes containing two vector bosons (hereafter referred to as dibosons) are normalized to the NLO predictions computed with MadGraph 5_amc@nlo, with the exception of the $\mathrm {W}$ $\mathrm {W}$ process, for which the NNLO prediction [43] is used.

All generated events are processed through the Geant4-based [44–46] CMS detector simulation and the standard CMS event reconstruction. Additional $\mathrm {p}\mathrm {p}$ collisions per bunch crossing (pileup) are included in the simulations. These simulate the effect of pileup in the events, with the same multiplicity distribution as that observed in data, i.e., about 23 simultaneous interactions, on average, per bunch crossing.

## Event reconstruction and selection

The selection targets events in which each $\mathrm {W}$ boson decays to a charged lepton and a neutrino. Data are selected online with single-lepton and dilepton triggers. The particle flow (PF) algorithm [9] is used for the reconstruction of final-state objects. The offline event selection is similar to the one described in Ref. [47]. At least one PF charged lepton candidate with $p_{\mathrm{T}} >25~\text {Ge}\text {V}$ and another one with $p_{\mathrm{T}} >20~\text {Ge}\text {V}$, both having $|\eta |<2.5$, are required. The two leptons must have opposite charges and an invariant mass $m(\ell ^\pm \ell ^\mp )>12~\text {Ge}\text {V}$. When extra leptons are present in the event, the dilepton candidate is built from the highest $p_{\mathrm{T}}$ leptons in the event. Events with $\mathrm {e}^\pm \mu ^\mp$ in the final state are used for the main analysis, while $\mathrm {e}^\pm \mathrm {e}^\mp$ and $\mu ^\pm \mu ^\mp$ events are used to derive the normalization of the DY background. The simulated events are corrected for the differences between data and simulation in the efficiencies of the trigger, lepton identification, and lepton isolation criteria. The corrections are derived with $\mathrm {Z}\rightarrow \mathrm {e}^\pm \mathrm {e}^\mp$ and $\mathrm {Z}\rightarrow \mu ^\pm \mu ^\mp$ events using the “tag-and-probe” method [48] and are parameterized as functions of the $p_{\mathrm{T}}$ and $\eta$ of the leptons.

Jets are clustered using the anti-$k_{\mathrm{T}}$ jet finding algorithm [49, 50] with a distance parameter of 0.4 and all the reconstructed PF candidates in the event. The charged hadron subtraction algorithm is used to mitigate the contribution from pileup to the jets [51]. At least two jets with $p_{\mathrm{T}} >30~\text {Ge}\text {V}$, $|\eta |<2.5$ and identified by a $\mathrm {b}$-tagging algorithm are required. The $\mathrm {b}$-tagging is based on a “combined secondary vertex” algorithm  [52] characterized by an efficiency of about 66%, corresponding to misidentification probabilities for light quark and $\mathrm {c}$ quark jets of 1.5 and 18%, respectively. A $p_{\mathrm{T}}$-dependent scale factor is applied to the simulations in order to reproduce the efficiency of this algorithm, as measured in data.

The reconstructed vertex with the largest value of summed physics-object $p_{\mathrm{T}} ^2$ is taken to be the primary $\mathrm {p}\mathrm {p}$ interaction vertex. The physics objects are the jets, clustered using the jet finding algorithm [49, 50] with the tracks assigned to the vertex as inputs, and the associated missing transverse momentum, $p_{\mathrm{T}} ^{\mathrm{miss}}$, taken as the negative vector sum of the $p_{\mathrm{T}}$ of those jets. The latter is defined as the magnitude of the negative vector sum of the momenta of all reconstructed PF candidates in an event, projected onto the plane perpendicular to the direction of the proton beams.

All backgrounds are estimated from simulation, with the exception of the DY background normalization. The latter is estimated making use of the so-called $R_{\mathrm{out/in}}$ method [53], in which events with same-flavor leptons are used to normalize the yield of $\mathrm {e}\mu$ pairs from DY production of $\tau$ lepton pairs. The normalization of the simulation is estimated from the number of events in the data within a 15$~\text {Ge}\text {V}$ window around the $\mathrm {Z}$ boson mass [53]. For $\mathrm {e}\mu$ events, we use the geometric mean of the scale factors determined for $\mathrm {e}\mathrm {e}$ and $\mu \mu$ events. With respect to the simulated predictions, a scale factor $1.3\pm 0.4$ is obtained from this method, with statistical and systematic uncertainties added in quadrature. The systematic uncertainty is estimated from the differences found in the scale factor for events with 0 or 1 $\mathrm {b}$-tagged jets, in the same-flavor channels.

We select a total of 52 645 $\mathrm {e}\mu$ events with an expected purity of 96%. The data agree with the expected yields within 2.2%, a value smaller than the uncertainty in the integrated luminosity alone, 2.5% [12]. The $\mathrm {t}$ $\mathrm {W}$ events are expected to constitute 90% of the total background.

In the simulation, the selection is mimicked at the particle level with the techniques described in Ref. [54]. Jets and leptons are defined at the particle level with the same conventions as adopted by the rivet framework [55]. The procedure ensures that the selections and definitions of the objects at particle level are consistent with those used in the rivet routines. A brief description of the particle-level definitions follows:

- prompt charged leptons (i.e., not produced as a result of hadron decays) are reconstructed as “dressed” leptons with nearby photon recombination using the anti-$k_{\mathrm{T}}$ algorithm with a distance parameter of 0.1;
- jets are clustered with the anti-$k_{\mathrm{T}}$ algorithm with a distance parameter of 0.4 using all particles remaining after removing both the leptons from the hard process and the neutrinos;
- the flavor of a jet is identified by including ${\mathrm {B}}$ hadrons in the clustering.

Using these definitions, the fiducial region of this analysis is specified by the same requirements that are applied offline (reconstruction level) for leptons and jets. Simulated events are categorized as fulfilling only the reconstruction-based, only the particle-based, or both selection requirements. If a simulated event passes only the reconstruction-level selection, it is considered in the “misidentified signal” category, i.e., it does not contribute to the fiducial region defined in the analysis and thus is considered as a background process. In the majority of the bins of each of the distributions analyzed, the fraction of signal events passing both the reconstruction- and particle-level selections is estimated to be about 80%, while the fraction of misidentified signal events is estimated to be less than 10%.

## Characterization of the underlying event

In order to isolate the UE activity in data, the contribution from both pileup and the hard process itself must be identified and excluded from the analysis. The contamination from pileup events is expected to yield soft particles in time with the hard process, as well as tails in the energy deposits from out-of-time interactions. The contamination from the hard process is expected to be associated with the two charged leptons and two $\mathrm {b}$ jets originating from the ${\mathrm {t}\overline{\mathrm {t}}}$ decay chain.

In order to minimize the contribution from these sources, we use the properties of the reconstructed PF candidates in each event. The track associated to the charged PF candidate is required to be compatible with originating from the primary vertex. This condition reduces to a negligible amount the contamination from pileup in the charged particle collection. A simple association by proximity in *z* with respect to the primary vertex of the event is expected to yield a pileup-robust, high-purity selection. For the purpose of this analysis all charged PF candidates are required to satisfy the following requirements:

- $$p_{\mathrm{T}} >900~\text {Me}\text {V} $$ and $$|\eta |<2.1$$;
- the associated track needs to be either used in the fit of the primary vertex or to be closer to it in *z* than with respect to other reconstructed vertices in the event.

**Fig. 1:**
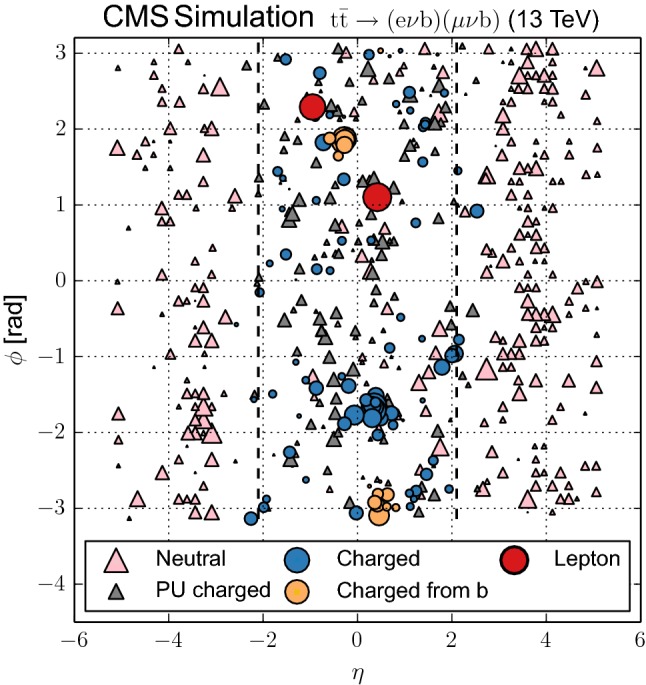
Distribution of all PF candidates reconstructed in a Pw+Py8 simulated ${\mathrm {t}\overline{\mathrm {t}}}$ event in the $\eta$–$\phi$ plane. Only particles with $p_{\mathrm{T}} >900~\text {Me}\text {V}$ are shown, with a marker whose area is proportional to the particle $p_{\mathrm{T}}$. The fiducial region in $\eta$ is represented by the dashed lines

After performing the selection of the charged PF candidates we check which ones have been used in the clustering of the two $\mathrm {b}$-tagged jets and which ones match the two charged lepton candidates within a $\varDelta R=\sqrt{\smash [b]{(\varDelta \eta )^2+(\varDelta \phi )^2}}=0.05$ cone, where $\phi$ is the azimuthal angle in radians. All PF candidates failing the kinematic requirements, being matched to another primary vertex in the event, or being matched to the charged leptons and $\mathrm {b}$-tagged jets, are removed from the analysis. The UE analysis proceeds by using the remaining charged PF candidates. Figure 1 shows, in a simulated ${\mathrm {t}\overline{\mathrm {t}}}$ event, the contribution from charged and neutral PF candidates, the charged component of the pileup, and the hard process. The charged PF candidates that are used in the study of the UE are represented after applying the selection described above.

Various characteristics, such as the multiplicity of the selected charged particles, the flux of momentum, and the topology or shape of the event have different sensitivity to the modeling of the recoil, the contribution from MPI and CR, and other parameters.

The first set of observables chosen in this analysis is related to the multiplicity and momentum flux in the event:

- charged-particle multiplicity: $N_{\mathrm{ch}}$;
- magnitude of the $p_{\mathrm{T}}$ of the charged particle recoil system: $|{\vec {p}}_{\mathrm{T}} |=|{\sum _{i=1}^{N_{\mathrm{ch}}}} \vec {p}_{\mathrm {T},i} |$;
- scalar sum of the $p_{\mathrm{T}}$ (or $p_z$) of charged particles: $\sum p_\mathrm{k}={\sum _{i=1}^{N_{\mathrm{ch}}}} |\vec {p}_{\mathrm {k},i} |$, where $\mathrm {k=T}$ or *z*;
- average $p_{\mathrm{T}}$ (or $p_z$) per charged particle: computed from the ratio between the scalar sum and the charged multiplicity: $\overline{p_{\mathrm{T}}}$ (or $\overline{p_z}$).

The second set of observables characterizes the UE shape and it is computed from the so-called linearized sphericity tensor [56, 57]:

1 $$\begin{aligned} S^{\mu \nu }= {\sum _{i=1}^{N_{\mathrm{ch}}}} p_i^\mu p_i^\nu / |p_i | \Big /~ {\sum _{i=1}^{N_{\mathrm{ch}}}} |p_i |, \end{aligned}$$

where the *i* index runs over the particles associated with the UE, as for the previous variables, and the $\mu$ and $\nu$ indices refer to one of the (*x*, *y*, *z*) components of the momentum of the particles. The eigenvalues ($\lambda _i$) of $S^{\mu \nu }$ are in decreasing order, i.e., with $\lambda _1$ the largest one, and are used to compute the following observables [58]:

- Aplanarity: $A=\frac{3}{2}\lambda _3$ measures the $p_{\mathrm{T}}$ component out of the event plane, defined by the two leading eigenvectors. Isotropic (planar) events are expected to have $A=1/2\,(0)$.
- Sphericity: $S=\frac{3}{2}(\lambda _2+\lambda _3)$ measures the $p_{\mathrm{T}} ^2$ with respect to the axis of the event. An isotropic (dijet) event is expected to have $S=1\,(0)$.
- ${C}=3(\lambda _1\lambda _2+\lambda _1\lambda _3+\lambda _2\lambda _3)$ identifies 3 jet events (tends to be 0 for dijet events).
- ${D}=27\lambda _1\lambda _2\lambda _3$ identifies 4 jet events (tends to be 0 otherwise).

Further insight can be gained by studying the evolution of the two sets of observables in different categories of the ${\mathrm {t}\overline{\mathrm {t}}}$ system kinematic quantities. The categories chosen below are sensitive to the recoil or the scale of the energy of the hard process, and are expected to be reconstructed with very good resolution. Additionally, these variables minimize the effect of migration of events between categories due to resolution effects.

**Fig. 2:**
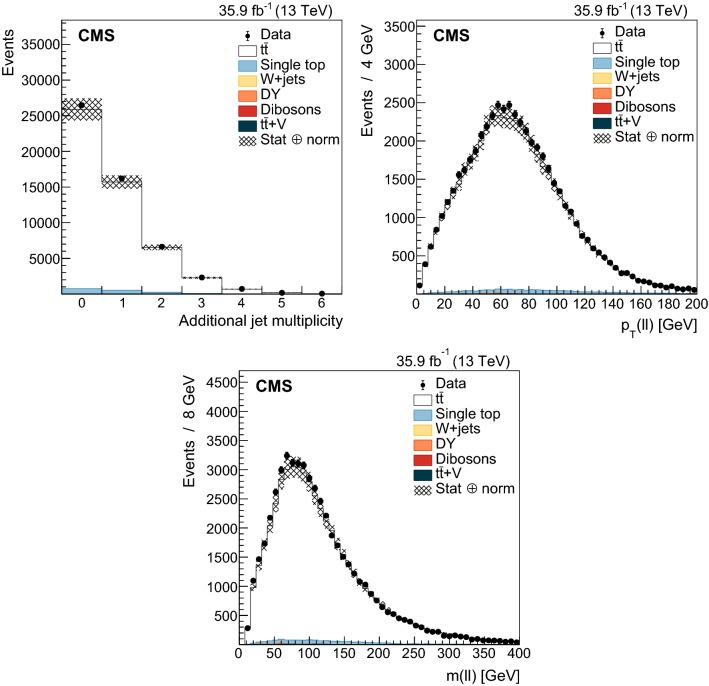
Distributions of the variables used to categorize the study of the UE. Upper left: multiplicity of additional jets ($p_{\mathrm{T}} >30~\text {Ge}\text {V}$). Upper right: $p_{\mathrm{T}} (\ell \ell )$. Lower: $m(\ell \ell )$. The distributions in data are compared to the sum of the expectations for the signal and backgrounds. The shaded band represents the uncertainty associated to the integrated luminosity and the theoretical value of the ${\mathrm {t}\overline{\mathrm {t}}}$ cross section

The dependence of the UE on the recoil system is studied in categories that are defined according to the multiplicity of additional jets with $p_{\mathrm{T}} >30~\text {Ge}\text {V}$ and $|\eta |<2.5$, excluding the two selected $\mathrm {b}$-tagged jets. The categories with 0, 1, or more than 1 additional jet are used for this purpose. The additional jet multiplicity is partially correlated with the charged-particle multiplicity and helps to factorize the contribution from ISR. The distribution of the number of additional jets is shown in Fig. 2 (upper left).

**Fig. 3:**
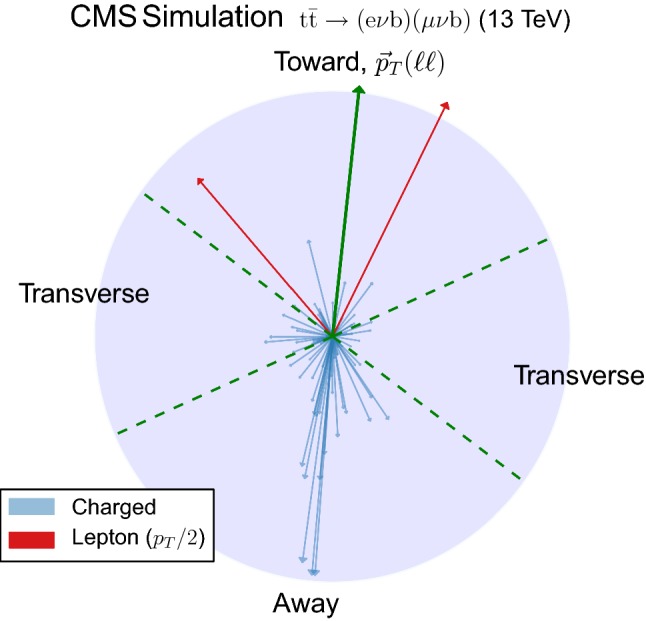
Display of the transverse momentum of the selected charged particles, the two leptons, and the dilepton pair in the transverse plane corresponding to the same event as in Fig. 1. The $p_{\mathrm{T}}$ of the particles is proportional to the length of the arrows and the dashed lines represent the regions that are defined relative to the ${\vec {p}}_{\mathrm{T}} (\ell \ell )$ direction. For clarity, the $p_{\mathrm{T}}$ of the leptons has been rescaled by a factor of 0.5

In addition to these categories, the transverse momentum of the dilepton system, ${\vec {p}}_{\mathrm{T}} (\ell \ell )$ , is used as it preserves some correlation with the transverse momentum of the ${\mathrm {t}\overline{\mathrm {t}}}$ system and, consequently, with the recoil of the system. The ${\vec {p}}_{\mathrm{T}} (\ell \ell )$ direction is used to define three regions in the transverse plane of each event. The regions are denoted as “transverse” ($60^\circ<|\varDelta \phi |<120^\circ$), “away” ($|\varDelta \phi |>120^\circ$), and “toward” ($|\varDelta \phi |<60^\circ$). Each reconstructed particle in an event is assigned to one of these regions, depending on the difference of their azimuthal angle with respect to the ${\vec {p}}_{\mathrm{T}} (\ell \ell )$ vector. Figure 3 illustrates how this classification is performed on a typical event. This classification is expected to enhance the sensitivity of the measurements to the contributions from ISR, MPI and CR in different regions. In addition, the magnitude, $p_{\mathrm{T}} (\ell \ell )$ , is used to categorize the events and its distribution is shown in Fig. 2 (upper right). The $p_{\mathrm{T}} (\ell \ell )$ variable is estimated with a resolution better than 3%.

Lastly, the dependence of the UE on the energy scale of the hard process is characterized by measuring it in different categories of the $m(\ell \ell )$ variable. This variable is correlated with the invariant mass of the ${\mathrm {t}\overline{\mathrm {t}}}$ system, but not with its $p_{\mathrm{T}}$. The $m(\ell \ell )$ distribution is shown in Fig. 2 (lower). A resolution better than 2% is expected in the measurement of $m(\ell \ell )$.

Although both $p_{\mathrm{T}} (\ell \ell )$ and $m(\ell \ell )$ are only partially correlated with the ${\mathrm {t}\overline{\mathrm {t}}}$ kinematic quantities, they are expected to be reconstructed with very good resolution. Because of the two escaping neutrinos, the kinematics of the ${\mathrm {t}\overline{\mathrm {t}}}$ pair can only be reconstructed by using the $p_{\mathrm{T}} ^{\mathrm{miss}}$ measurement, which has poorer experimental resolution when compared to the leptons. In addition, given that $p_{\mathrm{T}} ^{\mathrm{miss}}$ is correlated with the UE activity, as it stems from the balance of all PF candidates in the transverse plane, it could introduce a bias in the definition of the categories and the observables studied to characterize the UE. Hence the choice to use only dilepton-related variables.

## Corrections to the particle level

Inefficiencies of the track reconstruction due to the residual contamination from pileup, nuclear interactions in the tracker material, and accidental splittings of the primary vertex [59] are expected to cause a slight bias in the observables described above. The correction for these biases is estimated from simulation and applied to the data by means of an unfolding procedure.

At particle (generator) level, the distributions of the observables of interest are binned according to the resolutions expected from simulation. Furthermore, we require that each bin contains at least 2% of the total number of events. The migration matrix (*K*), used to map the reconstruction- to particle-level distributions, is constructed using twice the number of bins at the reconstruction level than the ones used at particle level. This procedure ensures almost diagonal matrices, which have a numerically stable inverse. The matrix is extended with an additional row that is used to count the events failing the reconstruction-level requirements, but found in the fiducial region of the analysis, i.e., passing the particle-level requirements. The inversion of the migration matrix is made using a Tikhonov regularization procedure [60], as implemented in the TUnfoldDensity package [61]. The unfolded distribution is found by minimizing a $\chi ^2$ function

2 $$\begin{aligned} \chi ^2=(y-K\lambda )^T V_{yy}^{-1} (y-K\lambda ) + \tau ^2 \Vert L(\lambda -\lambda _0)\Vert ^2, \end{aligned}$$

where *y* are the observations, $V_{yy}$ is an estimate of the covariance of *y* (calculated using the simulated signal sample), $\lambda$ is the particle-level expectation, $\Vert L(\lambda -\lambda _0)\Vert ^2$ is a penalty function (with $\lambda _0$ being estimated from the simulated samples), and $\tau >0$ is the so-called regularization parameter. The latter regulates how strongly the penalty term should contribute to the minimization of $\chi ^2$. In our setup we choose the function *L* to be the curvature, i.e., the second derivative, of the output distribution. The chosen value of the $\tau$ parameter is optimized for each distribution by minimizing its average global correlation coefficient [61]. Small values, i.e., $\tau <10^{-3}$, are found for all the distributions; the global correlation coefficients are around 50%. After unfolding, the distributions are normalized to unity.

The statistical coverage of the unfolding procedure is checked by means of pseudo-experiments based on independent Pw+Py8 samples. The pull of each bin in each distribution is found to be consistent with that of a standard normal distribution. The effect of the regularization term in the unfolding is checked in the data by folding the measured distributions and comparing the outcome to the originally-reconstructed data. In general the folded and the original distributions agree within 1–5% in each bin, with the largest differences observed in bins with low yield.

## Systematic uncertainties

The impact of different sources of uncertainty is evaluated by unfolding the data with alternative migration matrices, which are obtained after changing the settings in the simulations as explained below. The effect of a source of uncertainty in non-fiducial ${\mathrm {t}\overline{\mathrm {t}}}$ events is included in this estimate, by updating the background prediction. The observed bin-by-bin differences are used as estimates of the uncertainty. The impact of the uncertainty in the background normalization is the only exception to this procedure, as detailed below. The covariance matrices associated to each source of uncertainty are built using the procedure described in detail in [62]. In case several sub-contributions are used to estimate a source of uncertainty, the corresponding differences in each bin are treated independently, symmetrized, and used to compute individual covariance matrices, which preserve the normalization. Variations on the event yields are fully absorbed by normalizing the measured cross sections. Thus, only the sources of uncertainty that yield variations in the shapes have a non-negligible impact.

### Experimental uncertainties

The following experimental sources of uncertainty are considered:

Pileup: Although pileup is included in the simulation, there is an intrinsic uncertainty in modeling its multiplicity. An uncertainty of $\pm 4.6\%$ in the inelastic $\mathrm {p}\mathrm {p}$ cross section is used and propagated to the event weights [63].
Trigger and selection efficiency: The scale factors used to correct the simulation for different trigger and lepton selection efficiencies in data and simulation are varied up or down, according to their uncertainty. The uncertainties in the muon track and electron reconstruction efficiencies are included in this category and added in quadrature.
Lepton energy scale: The corrections applied to the electron energy and muon momentum scales are varied separately, according to their uncertainties. The corrections and uncertainties are obtained using methods similar to those described in Refs. [64, 65]. These variations lead to a small migration of events between the different $p_{\mathrm{T}} (\ell \ell )$ or $m(\ell \ell )$ categories used in the analysis.
Jet energy scale: A $p_{\mathrm{T}}$- and $\eta$-dependent parameterization of the jet energy scale is used to vary the calibration of the jets in the simulation. The corrections and uncertainties are obtained using methods similar to those described in Ref. [51]. The effect of these variations is similar to that described for the lepton energy scale uncertainty; in this case the migration of events occurs between different jet multiplicity categories.
Jet energy resolution: Each jet is further smeared up or down depending on its $p_{\mathrm{T}}$ and $\eta$, with respect to the central value measured in data. The difference with respect to data is measured using methods similar to those described in Ref. [51]. The main effect induced in the analysis from altering the jet energy resolution is similar to that described for the jet energy scale uncertainty.
$\mathrm {b}$ tagging and misidentification efficiencies:
The scale factors used to correct for the difference in performance between data and simulation are varied according to their uncertainties and depending on the flavor of the jet [52]. The main effect of this variation is to move jets into the candidate $\mathrm {b}$ jets sample or remove them from it.
Background normalization: The impact of the uncertainty in the normalization of the backgrounds is estimated by computing the difference obtained with respect to the nominal result when these contributions are not subtracted from data. This difference is expected to cover the uncertainty in the normalization of the main backgrounds, i.e., DY and the $\mathrm {t}$ $\mathrm {W}$ process, and the uncertainty in the normalization of the ${\mathrm {t}\overline{\mathrm {t}}}$ events that are expected to pass the reconstruction-level requirements but fail the generator-level ones. The total expected background contribution is at the level of 8–10%, depending on the bin. The impact from this uncertainty is estimated to be $<5\%$.
Tracking reconstruction efficiency: The efficiency of track reconstruction is found to be more than 90%. It is monitored using muon candidates from $\mathrm {Z}\rightarrow \mu ^+ \mu ^-$ decays, and the ratio of the four-body final $\mathrm {D}^0 \rightarrow \mathrm {K}^-\pi ^+ \pi ^- \pi ^+$ decay to the two-body $\mathrm {D}^0 \rightarrow \mathrm {K}^-\pi ^+$ decay. The latter is used to determine a data-to-simulation scale factor ($SF_{\mathrm{trk}}$) as a function of the pseudorapidity of the tracks, and for different periods of the data taking used in this analysis. The envelope of the $SF_{\mathrm{trk}}$ values, with uncertainties included, ranges from 0.89 to 1.17 [66], and it provides an adequate coverage for the residual variations observed in the charged-particle multiplicity between different data taking periods. The impact of the variation of $SF_{\mathrm{trk}}$ by its uncertainty is estimated by using the value of $|1-SF_{\mathrm{trk}} |$ for the probability to remove a reconstructed track from the event or to promote an unmatched generator-level charged particle to a reconstructed track, depending on whether $SF_{\mathrm{trk}}<1$ or $>1$, respectively. Different migration matrices, reflecting the different tracking efficiencies obtained from varying the uncertainty in $SF_{\mathrm{trk}}$, are obtained by this method and used to unfold the data. Although the impact is nonnegligible on variables such as $N_{\mathrm{ch}}$ or $\sum p_{\mathrm{T}}$ , it has very small impact ($<1\%$) on variables such as $\overline{p_{\mathrm{T}}}$ and $\overline{p_z}$.

### Theoretical uncertainties

The following theoretical uncertainties are considered:

Scale choices: $\mu _\mathrm{R}$ and $\mu _\mathrm{F}$ are varied individually in the ME by factors between 0.5 and 2, excluding the extreme cases $\mu _\mathrm{R}/\mu _\mathrm{F}=\mu (2,0.5)$ and $\mu (0.5,2)$, according to the prescription described in Refs. [67, 68].
Resummation scale and $\alpha _S$ used in the parton shower:
In powheg, the real emission cross section is scaled by a damping function, parameterized by the so-called $h_{\mathrm{damp}}$ variable [13–15]. This parameter controls the ME-PS matching and regulates the high-$p_{\mathrm{T}}$ radiation by reducing real emissions generated by powheg with a factor of $h_{\mathrm{damp}}^2/(p_{\mathrm{T}} ^2+h_{\mathrm{damp}}^2)$. In the simulation used to derive the migration matrices, $h_{\mathrm{damp}}=1.58~m_\mathrm {t}$ and the uncertainty in this value is evaluated by changing it by +42 or -37%, a range that is determined from the jet multiplicity measurements in ${\mathrm {t}\overline{\mathrm {t}}}$ at $\sqrt{s}=8~\text {Te}\text {V}$ [20]. Likewise, the uncertainty associated with the choice of $\alpha _S ^{\mathrm{ISR}}(M_\mathrm {Z})=0.1108$ for space-like and $\alpha _S ^{\mathrm{FSR}}(M_\mathrm {Z})=0.1365$ for time-like showers in the CUETP8M2T4 tune is evaluated by varying the scale at which it is computed, $M_\mathrm {Z}$, by a factor of 2 or 1/2.
UE model: The dependence of the migration matrix on the UE model assumed in the simulation is tested by varying the parameters that model the MPI and CR in the range of values corresponding to the uncertainty envelope associated to the CUETP8M2T4 tune. The uncertainty envelope has been determined using the same methods as described in Ref. [19]. In the following, these will be referred to as UE up/down variations. The dependence on the CR model is furthermore tested using other models besides the nominal one, which is the MPI-based CR model where the ${\mathrm {t}\overline{\mathrm {t}}}$ decay products are excluded from reconnections to the UE. A dedicated sample where the reconnections to resonant decay products are enabled (hereafter designated as ERDon) is used to evaluate possible differences in the unfolded results. In addition, alternative models for the CR are tested. One sample utilizing the “gluon move” model [5], in which gluons can be moved to another string, and another utilizing the “QCD-based” model with string formation beyond LO [32] are used for this purpose. In both samples, the reconnections to the decay resonant processes are enabled. The envelope of the differences is considered as a systematic uncertainty.
$$\mathrm {t}$$ quark $$\mathbf p_{\mathrm{T}} $$:
The effect of reweighting of the simulated $\mathrm {t}$ quark $p_{\mathrm{T}}$ ($p_{\mathrm{T}} (\mathrm {t})$) distribution to match the one reconstructed from data [69, 70] is added as an additional uncertainty. This has the most noticeable effect on the fraction of events that do not pass the reconstruction-level requirements and migrate out of the fiducial phase space.
$$\mathrm {t}$$ quark mass:
An additional uncertainty is considered, related to the value of $m_\mathrm {t}=172.5~\text {Ge}\text {V}$ used in the simulations, by varying this parameter by $\pm 0.5~\text {Ge}\text {V}$ [71].

Any possible uncertainty from the choice of the hadronization model is expected to be significantly smaller than the theory uncertainties described above. This has been explicitly tested by comparing the results at reconstruction level and after unfolding the data with the Pw+Py8 and Pw+Hw++ migration matrices. The latter relies on a different hadronization model, but it folds other modelling differences such as the underlying event tune or the parton shower as well. Thus it can only be used as a test setup to validate the measurement.

### Summary of systematic uncertainties

The uncertainties on the measurement of the normalized differential cross sections are dominated by the systematic uncertainties, although in some bins of the distributions the statistical uncertainties are a large component. The experimental uncertainties have, in general, small impact; the most relevant are the tracking reconstruction efficiency for the $N_{\mathrm{ch}}$, $\sum p_{\mathrm{T}}$ , $\sum p_{z}$ , and $|{\vec {p}}_{\mathrm{T}} |$ observables. Other observables are affected at a sub-percent level by this uncertainty. Theory uncertainties affect the measurements more significantly, a fact that underlines the need of better tuning of the model parameters.

Event shape observables are found to be the most robust against this uncertainty, while $\sum p_{\mathrm{T}}$ , $\sum p_{z}$ , and $|{\vec {p}}_{\mathrm{T}} |$ are the ones that suffer more from it. Other sources of theoretical uncertainty typically have a smaller effect.

To further illustrate the impact of different sources on the observables considered, we list in Table 2 the uncertainties on the average of each observable. In the table, only systematic uncertainties that impact the average of one of the observables by at least 0.5% are included. The total uncertainty on the average of a given quantity ranges from 1 to 8%, and hence the comparison with model predictions can be carried out in a discrete manner.

**Table 2:** Uncertainties affecting the measurement of the average of the UE observables. The values are expressed in % and the last row reports the quadratic sum of the individual contributions

Source	% Uncertainty									
	$N_{\mathrm{ch}}$	$\sum p_{\mathrm{T}}$	$\sum p_{z}$	$\overline{p_{\mathrm{T}}}$	$\overline{p_z}$	$|{\vec {p}}_{\mathrm{T}} |$	*S*	*A*	*C*	*D*
Statistical	0.1	0.2	0.3	0.2	0.2	0.3	0.1	0.1	0.1	0.1
*Experimental*										
Background	1.2	1.6	1.8	0.4	0.7	1.6	0.4	0.7	0.3	0.7
Tracking eff.	4.4	4.2	4.9	0.8	0.4	4.0	0.4	0.6	0.2	0.6
*Theory*										
$\mu _\mathrm{R}/\mu _\mathrm{F}$	0.5	0.8	1.0	0.3	0.3	1.0	0.1	0.1	0.1	0.2
Resummation scale	0.2	0.8	0.5	1.1	0.2	1.6	0.8	0.4	0.2	0.7
$\alpha _S ^{\mathrm{FSR}}(M_\mathrm {Z})$	0.5	0.7	0.7	0.8	1.7	0.7	0.2	1.0	0.2	1.2
$\alpha _S ^{\mathrm{ISR}}(M_\mathrm {Z})$	0.1	0.3	1.1	1.2	0.7	0.4	0.2	0.5	0.1	1.3
UE model	0.1	0.1	0.2	1.0	0.4	0.5	0.2	0.2	0.1	0.9
$m_\mathrm {t}$	0.4	0.7	1.5	0.6	0.9	0.5	0.1	0.1	0.1	0.7
$p_{\mathrm{T}} (\mathrm {t})$	1.4	4.4	4.5	2.8	2.1	6.7	0.2	0.5	0.2	0.3
Total	4.9	6.5	7.3	3.7	3.1	8.2	1.1	1.6	0.6	2.4

## Results

### Inclusive distributions

**Fig. 4:**
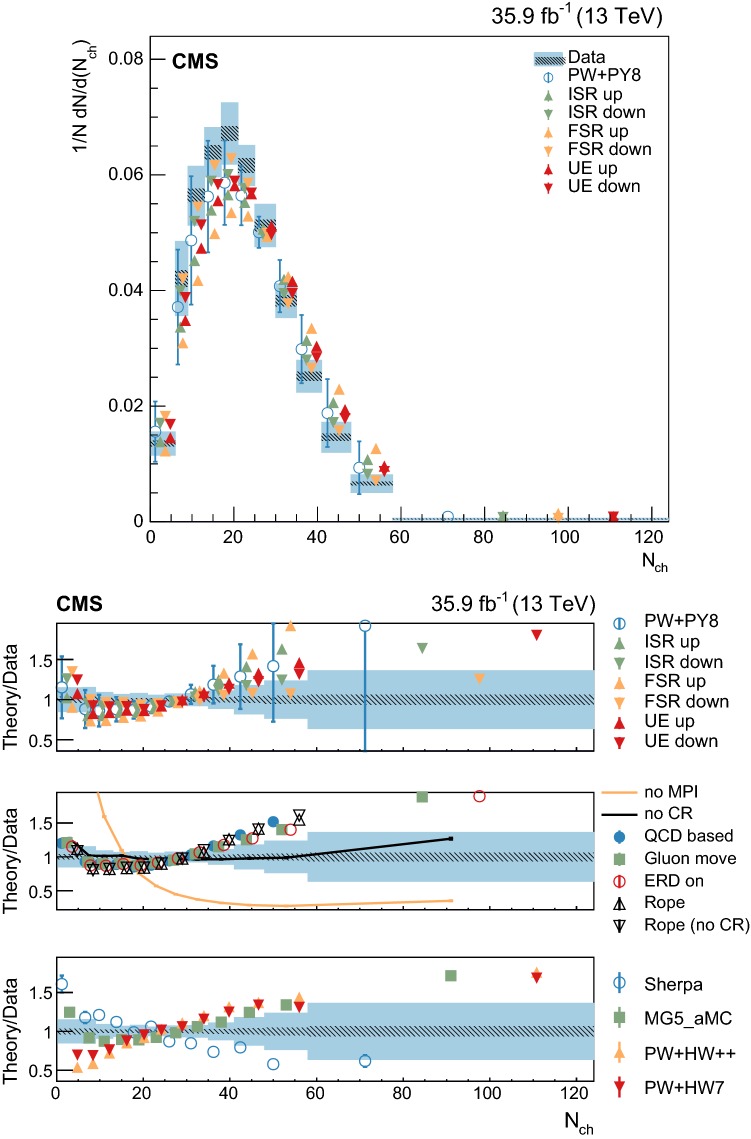
The normalized differential cross section as a function of $N_{\mathrm{ch}}$ is shown on the upper panel. The data (colored boxes) are compared to the nominal Pw+Py8 predictions and to the expectations obtained from varied $\alpha _S ^{\mathrm{ISR}}(M_\mathrm {Z})$ or $\alpha _S ^{\mathrm{FSR}}(M_\mathrm {Z})$ Pw+Py8 setups (markers). The different panels on the lower display show the ratio between each model tested (see text) and the data. In both cases the shaded (hatched) band represents the total (statistical) uncertainty of the data, while the error bars represent either the total uncertainty of the Pw+Py8 setup, computed as described in the text, or the statistical uncertainty of the other MC simulation setups

**Fig. 5:**
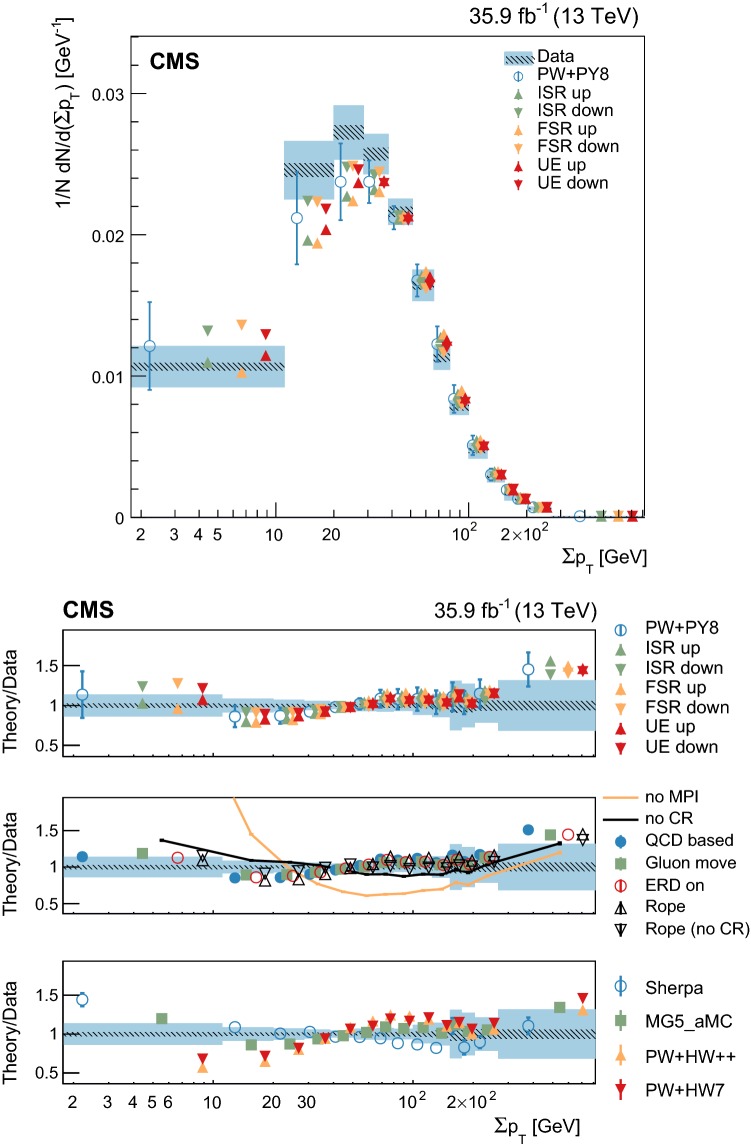
Normalized differential cross section as function of $\sum p_{\mathrm{T}}$ , compared to the predictions of different models. The conventions of Fig. 4 are used

**Fig. 6:**
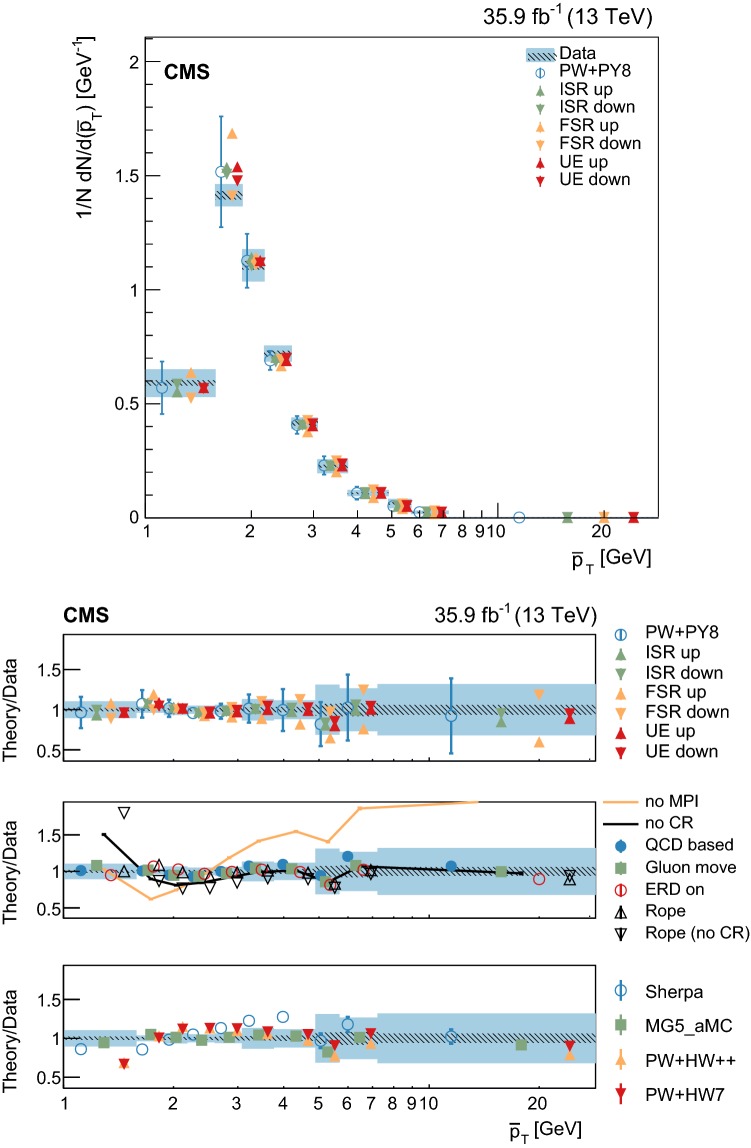
Normalized differential cross section as function of $\overline{p_{\mathrm{T}}}$ , compared to the predictions of different models. The conventions of Fig. 4 are used

**Fig. 7:**
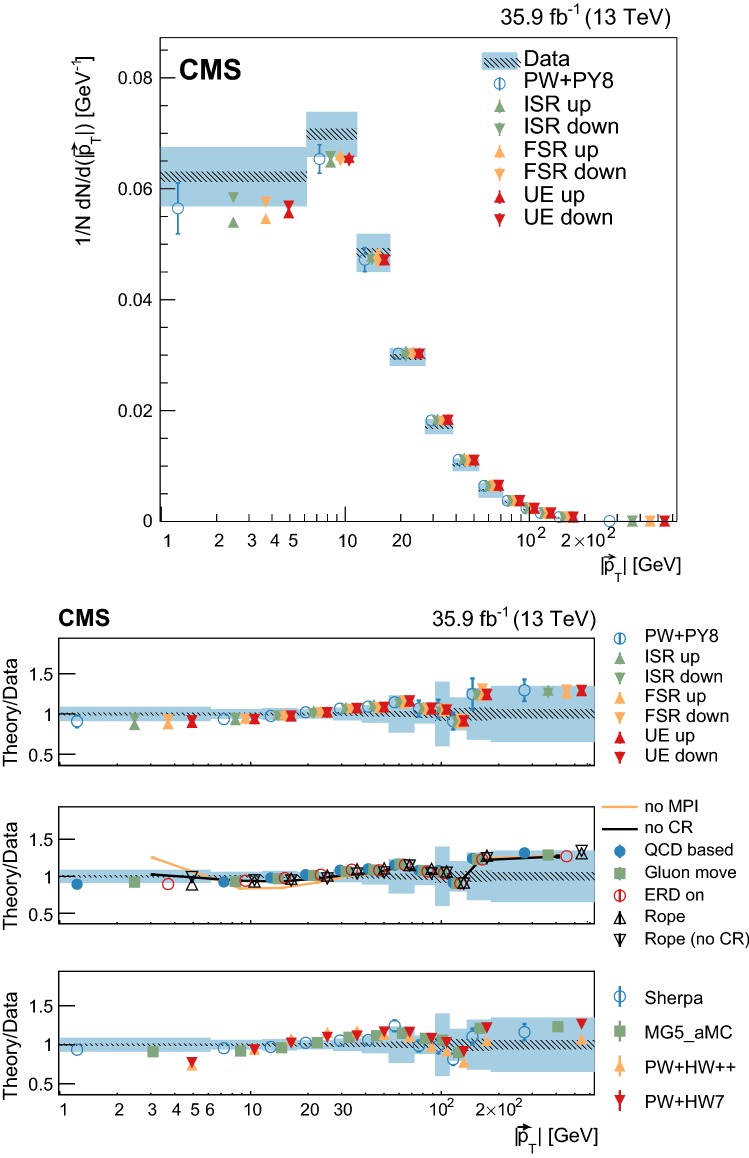
Normalized differential cross section as function of $|{\vec {p}}_{\mathrm{T}} |$ , compared to the predictions of different models. The conventions of Fig. 4 are used

**Fig. 8:**
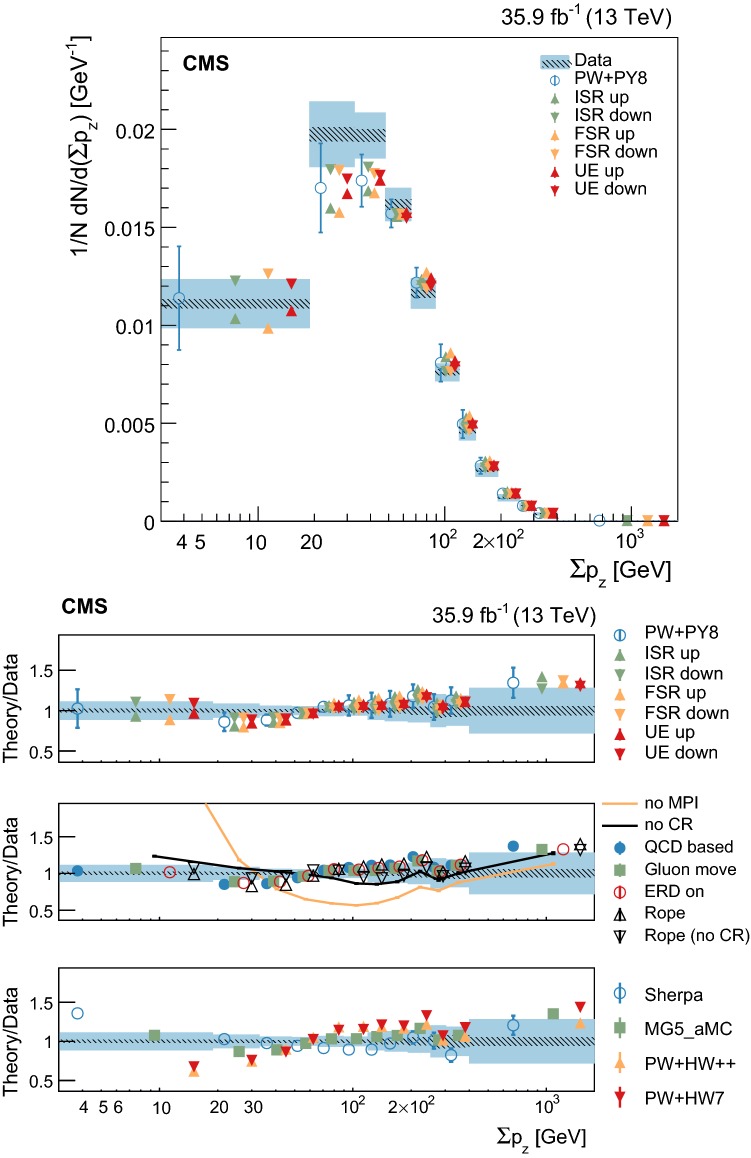
Normalized differential cross section as function of $\sum p_{z}$ , compared to the predictions of different models. The conventions of Fig. 4 are used

**Fig. 9:**
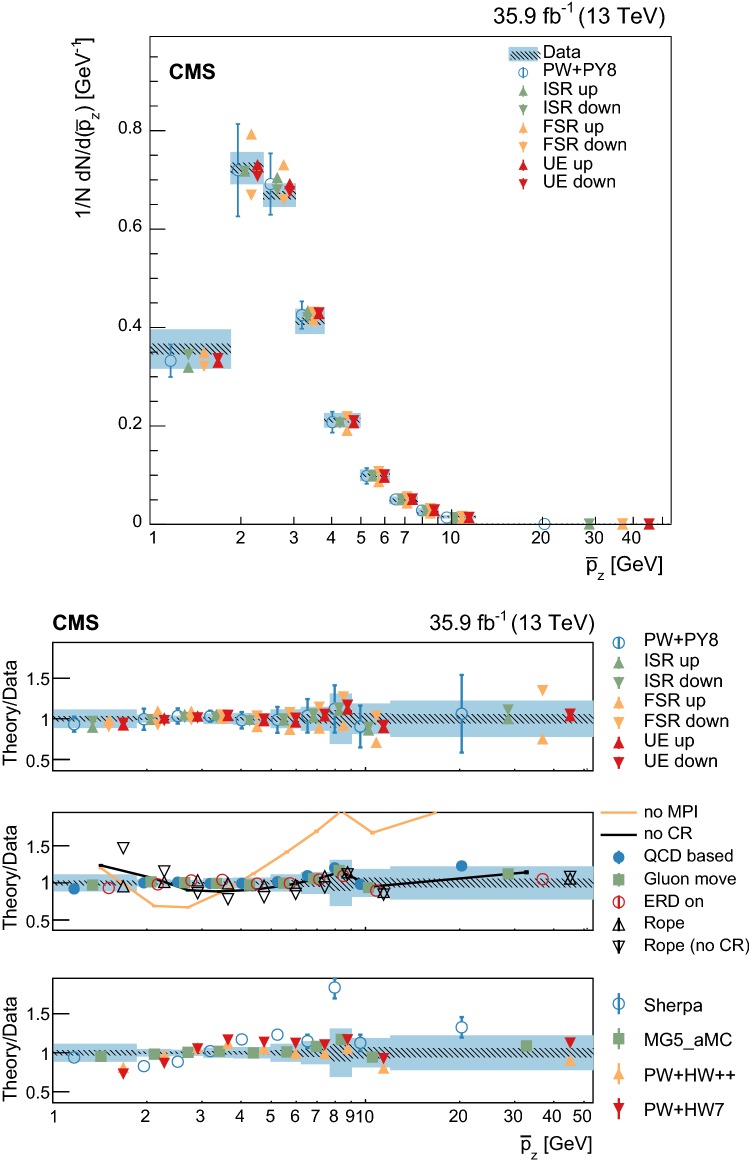
Normalized differential cross section as function of $\overline{p_z}$ , compared to the predictions of different models. The conventions of Fig. 4 are used

**Fig. 10:**
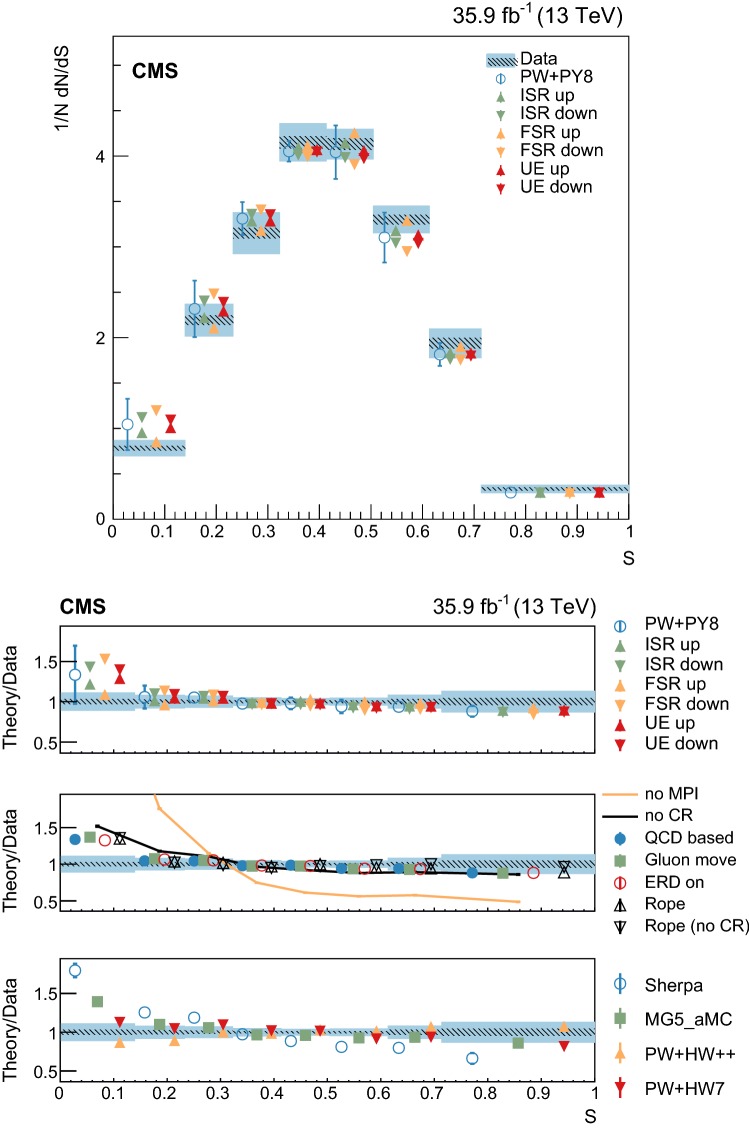
Normalized differential cross section as function of the sphericity variable, compared to the predictions of different models. The conventions of Fig. 4 are used

**Fig. 11:**
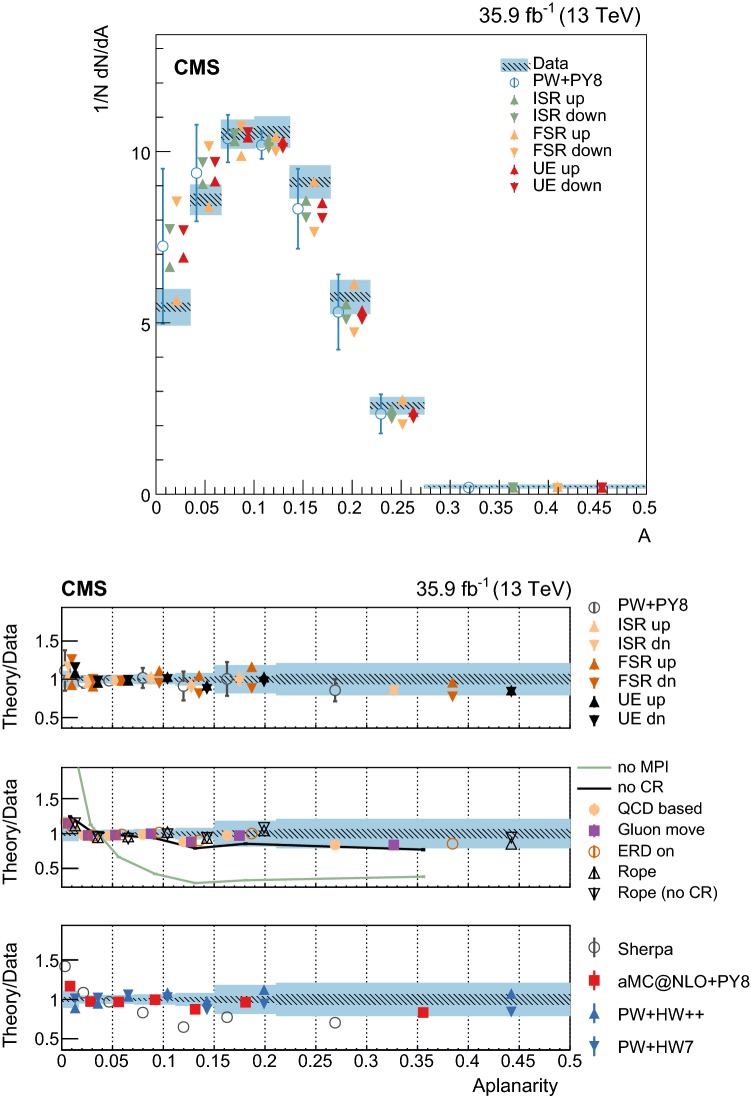
Normalized differential cross section as function of the aplanarity variable, compared to the predictions of different models. The conventions of Fig. 4 are used

**Fig. 12:**
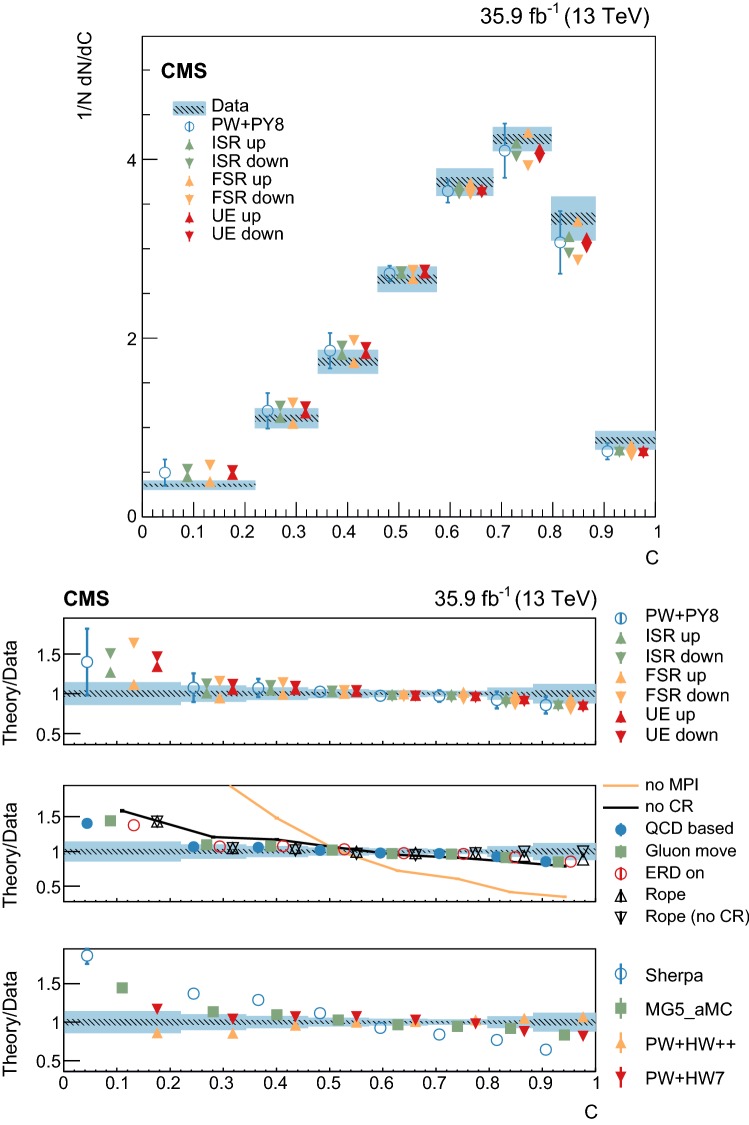
Normalized differential cross section as function of the *C* variable, compared to the predictions of different models. The conventions of Fig. 4 are used

**Fig. 13:**
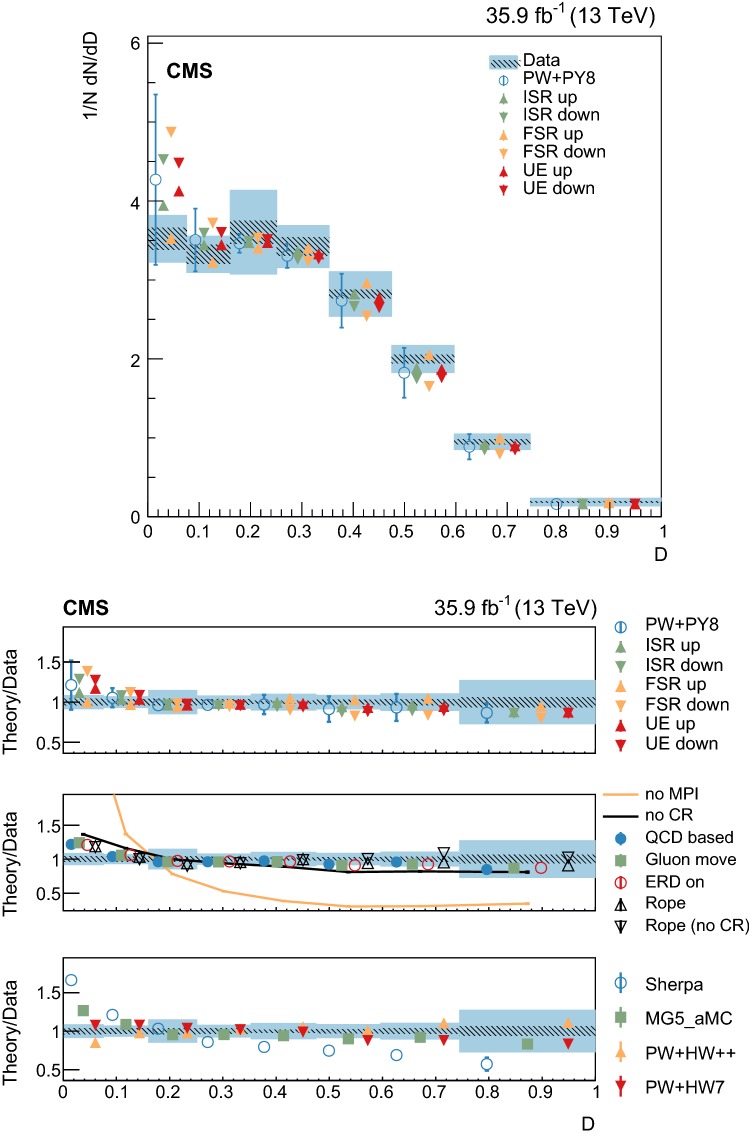
Normalized differential cross section as function of the *D* variable, compared to the predictions of different models. The conventions of Fig. 4 are used

The normalized differential cross sections measured as functions of $N_{\mathrm{ch}}$, $\sum p_{\mathrm{T}}$, $\overline{p_{\mathrm{T}}}$, $|{\vec {p}}_{\mathrm{T}} |$, $\sum p_{z}$, $\overline{p_z}$, sphericity, aplanarity, *C*, and *D* are shown in Figs. 4, 5, 6, 7, 8, 9, 10, 11, 12 and 13, respectively. The distributions are obtained after unfolding the background-subtracted data and normalizing the result to unity. The result is compared to the simulations, whose settings are summarized in Table 1 and in the appendix. For the predictions, the statistical uncertainty is represented as an error bar. In the specific case of the Pw+Py8 setup, the error bar represents the envelope obtained by varying the main parameters of the CUETP8M2T4 tune, according to their uncertainties. The envelope includes the variation of the CR model, $\alpha _S ^{\mathrm{ISR}}(M_\mathrm {Z})$, $\alpha _S ^{\mathrm{FSR}}(M_\mathrm {Z})$, the $h_{\mathrm{damp}}$ parameter, and the $\mu _\mathrm{R}/\mu _\mathrm{F}$ scales at the ME level. Thus, the uncertainty band represented for the Pw+Py8 setup should be interpreted as the theory uncertainty in that prediction. For each distribution we give, in addition, the ratio between different predictions and the data.

In ${\mathrm {t}\overline{\mathrm {t}}}$ events the UE contribution is determined to have typically $\mathcal {O}(20)$ charged particles with $\overline{p_{\mathrm{T}}} \sim \overline{p_z} \approx 2~\text {Ge}\text {V}$, vectorially summing to a recoil of about 10$~\text {Ge}\text {V}$. The distribution of the UE activity is anisotropic (as the sphericity is $<1$), close to planar (as the aplanarity peaks at low values of $\approx$0.1), and peaks at around 0.75 in the *C* variable, which identifies three-jet topologies. The *D* variable, which identifies the four-jet topology, is instead found to have values closer to 0. The three-prong configuration in the energy flux of the UE described by the *C* variable can be identified with two of the eigenvectors of the linearized sphericity tensor being correlated with the direction of the $\mathrm {b}$-tagged jets, and the third one being determined by energy conservation. When an extra jet with $p_{\mathrm{T}} >30~\text {Ge}\text {V}$ is selected, we measure a change in the profile of the event shape variables, with average values lower by 20–40% with respect to the distributions in which no extra jet is found. Thus when an extra jet is present, the event has a dijet-like topology instead of an isotropic shape.

The results obtained with pythia8 for the parton shower simulation show negligible dependence on the ME generator with which it is interfaced, i.e., Pw+Py8 and MG5_aMC yield similar results. In all distributions the contribution from MPI is strong: switching off this component in the simulation has a drastic effect on the predictions of all the variables analyzed. Color reconnection effects are more subtle to identify in the data. In the inclusive distributions, CR effects are needed to improve the theory accuracy for $\overline{p_{\mathrm{T}}} <3~\text {Ge}\text {V}$ or $\overline{p_z} <5~\text {Ge}\text {V}$. The differences between the CR models tested (as discussed in detail in Sect. 3) are nevertheless small and almost indistinguishable in the inclusive distributions. In general the Pw+Py8 setup is found to be in agreement with the data, when the total theory uncertainty is taken into account. In most of the distributions it is the variation of $\alpha _S ^{\mathrm{FSR}}(M_\mathrm {Z})$ that dominates the theory uncertainty, as this variation leads to the most visible changes in the UE. The other parton shower setups tested do not describe the data as accurately, but they were not tuned to the same level of detail as Pw+Py8. The Pw+Hw++ and Pw+Hw7-based setups show distinct trends with respect to the data from those observed in any of the pythia8-based setups. While describing fairly well the UE event shape variables, herwig++ and herwig 7 disagree with the $N_{\mathrm{ch}}$, $\overline{p_{\mathrm{T}}}$ , and $\overline{p_z}$ measurements. The sherpa predictions disagree with data in most of the observables.

For each distribution the level of agreement between theory predictions and data is quantified by means of a $\chi ^2$ variable defined as:

3 $$\begin{aligned} \chi ^2= \sum _{i,j=1}^{n}\delta y_i\,(\text {Cov}^{-1})_{ij}\,\delta y_j, \end{aligned}$$

where $\delta y_i$ ($\delta y_j$) are the differences between the data and the model in the *i*-th (*j*-th) bin; here *n* represents the total number of bins, and $\text {Cov}^{-1}$ is the inverse of the covariance matrix. Given that the distributions are normalized, the covariance matrix becomes invertible after removing its first row and column. In the calculation of Eq. 3 we assume that the theory uncertainties are uncorrelated with those assigned to the measurements. Table 3 summarizes the values obtained for the main models with respect to each of the distributions shown in Figs. 4, 5, 6, 7, 8, 9, 10, 11, 12 and 13. The values presented in the table quantify the level of agreement of each model with the measurements. Low $\chi ^2$ per number of degrees of freedom (dof) values are obtained for the Pw+Py8 setup when all the theory uncertainties of the model are taken into account, in particular for the event shape variables. This indicates that the theory uncertainty envelope is conservative.

**Table 3:** Comparison between the measured distributions at particle level and the predictions of different generator setups. We list the results of the $\chi ^2$ tests together with dof. For the comparison no uncertainties in the predictions are taken into account, except for the Pw+Py8 setup for which the comparison including the theoretical uncertainties is quoted separately in parenthesis

Observable	$\chi ^2/$dof				
	Pw+Py8	Pw+Hw++	Pw+Hw7	MG5_aMC	sherpa
$N_{\mathrm{ch}}$	30/11 (15/11)	33/11	17/11	34/11	95/11
$\sum p_{\mathrm{T}}$	24/13 (13/13)	129/13	56/13	30/13	37/13
$\sum p_{z}$	8/11 (4/11)	34/11	20/11	9/11	18/11
$\overline{p_{\mathrm{T}}}$	12/9 (1/9)	40/9	56/9	6/9	56/9
$\overline{p_z}$	2/9 (1/9)	9/9	32/9	1/9	36/9
$|{\vec {p}}_{\mathrm{T}} |$	17/11 (7/11)	102/11	49/11	20/11	34/11
*S*	29/7 (3/7)	7/7	17/7	36/7	194/7
*A*	18/7 (1/7)	8/7	13/7	26/7	167/7
*C*	34/7 (4/7)	7/7	27/7	38/7	187/7
*D*	7/7 (1/7)	5/7	8/7	11/7	83/7

### Profile of the UE in different categories

**Fig. 14:**
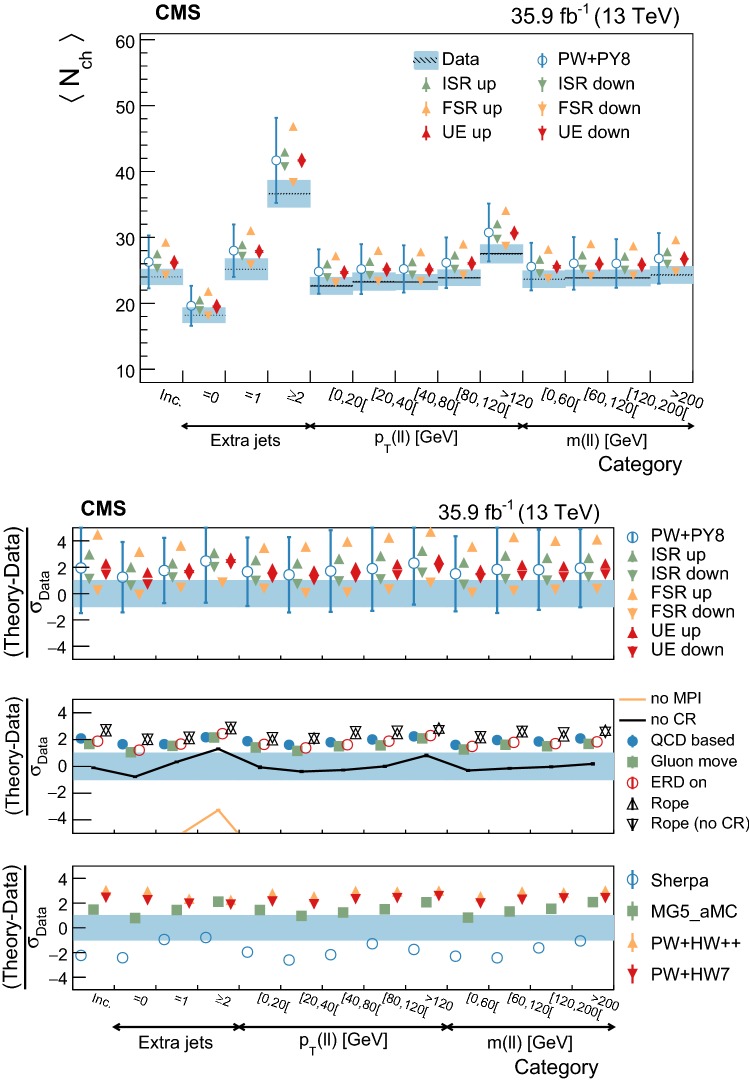
Average $N_{\mathrm{ch}}$ in different event categories. The mean observed in data (boxes) is compared to the predictions from different models (markers), which are superimposed in the upper figure. The total (statistical) uncertainty of the data is represented by a shaded (hatched) area and the statistical uncertainty of the models is represented with error bars. In the specific case of the Pw+Py8 model the error bars represent the total uncertainty (see text). The lower figure displays the pull between different models and the data, with the different panels corresponding to different sets of models. The bands represent the interval where $|\text {pull} |<1$. The error bar for the Pw+Py8 model represents the range of variation of the pull for the different configurations described in the text

**Fig. 15:**
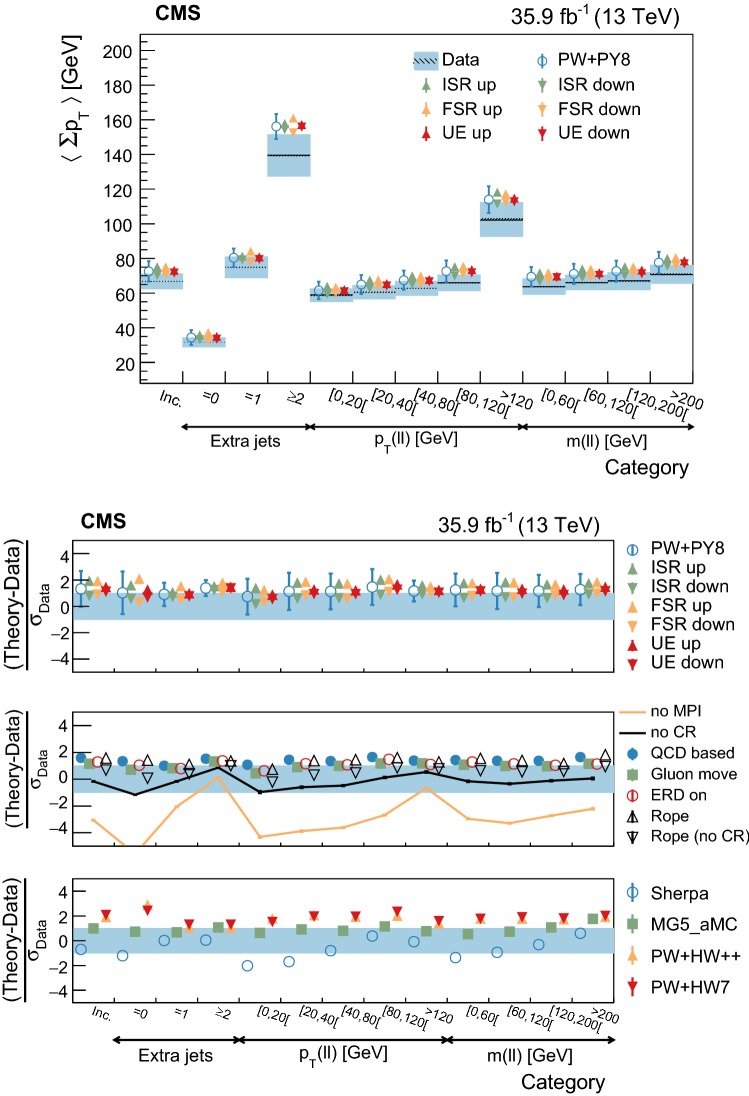
Average $\sum p_{\mathrm{T}}$ in different event categories. The conventions of Fig. 14 are used

**Fig. 16:**
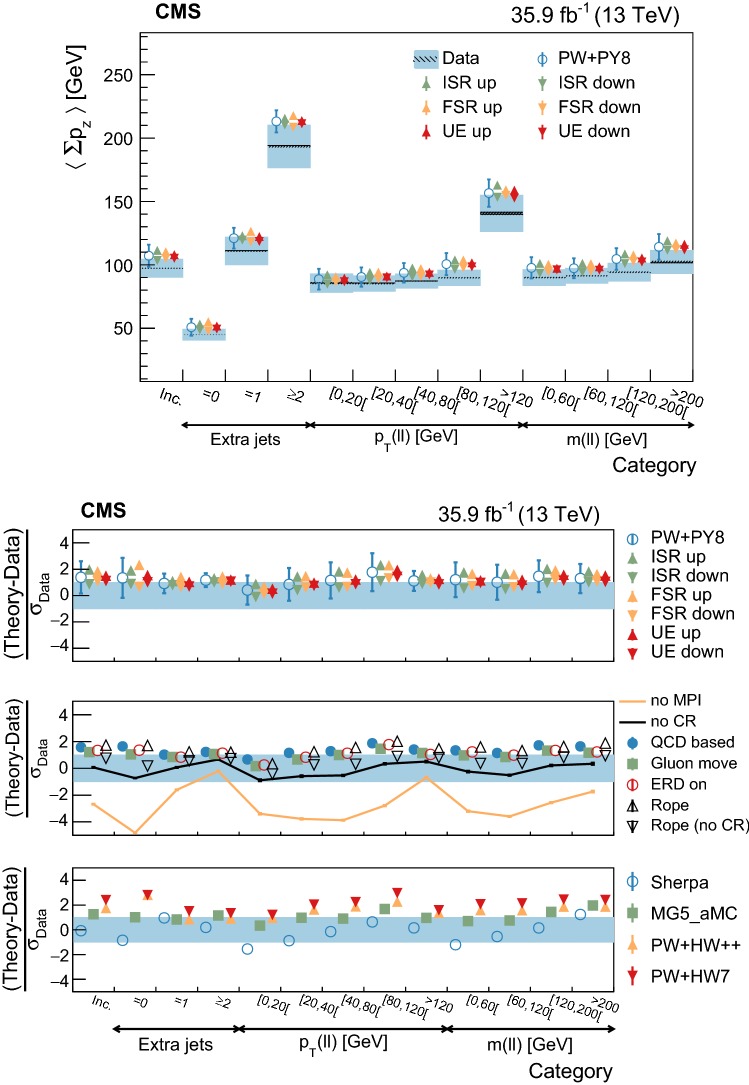
Average $\sum p_{z}$ in different categories. The conventions of Fig. 14 are used

**Fig. 17:**
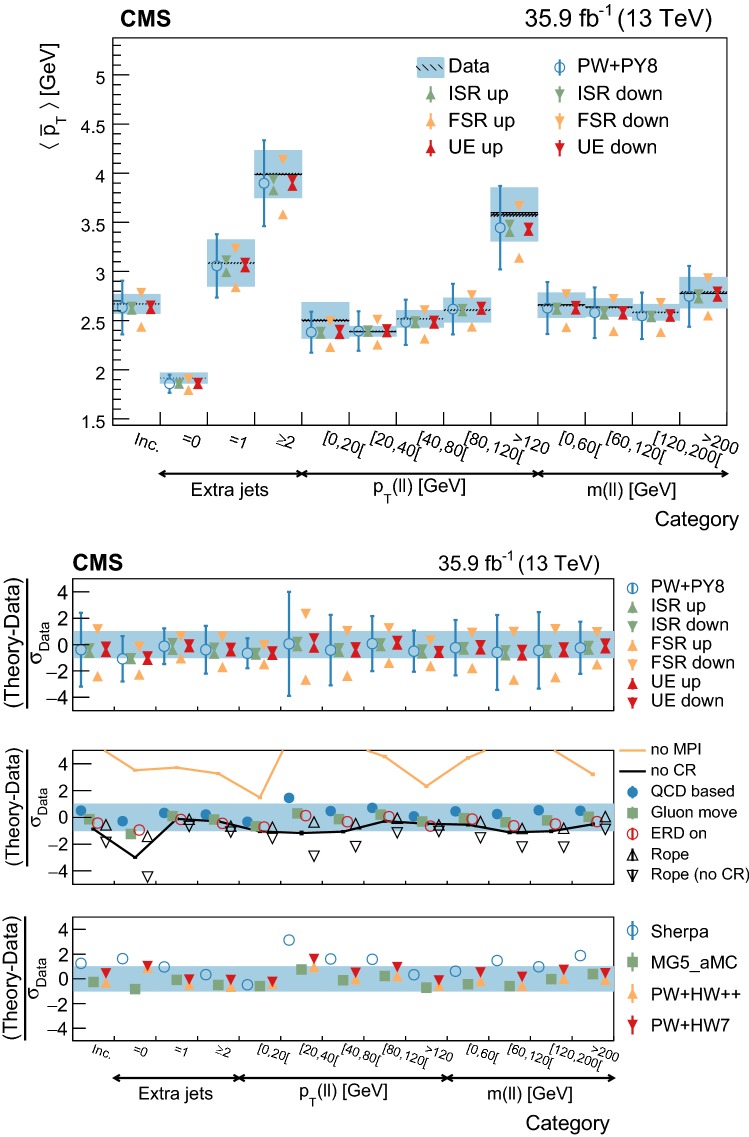
Average $\overline{p_{\mathrm{T}}}$ in different categories. The conventions of Fig. 14 are used

**Fig. 18:**
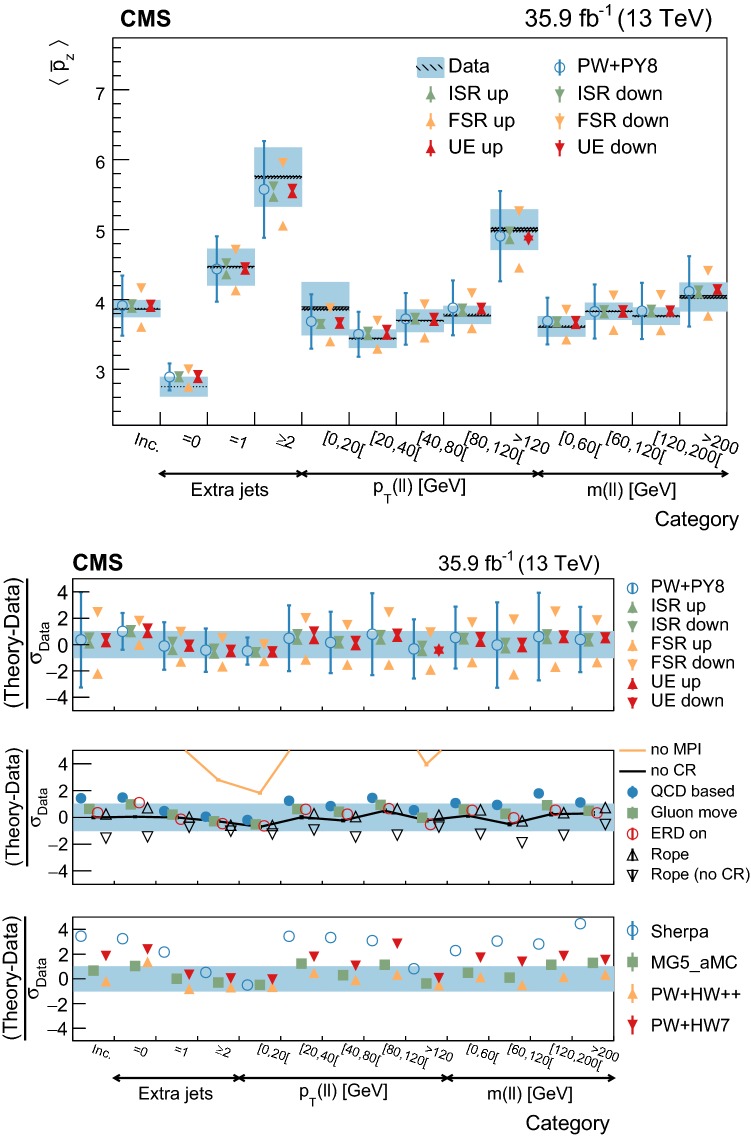
Average $\overline{p_z}$ in different categories. The conventions of Fig. 14 are used

**Fig. 19:**
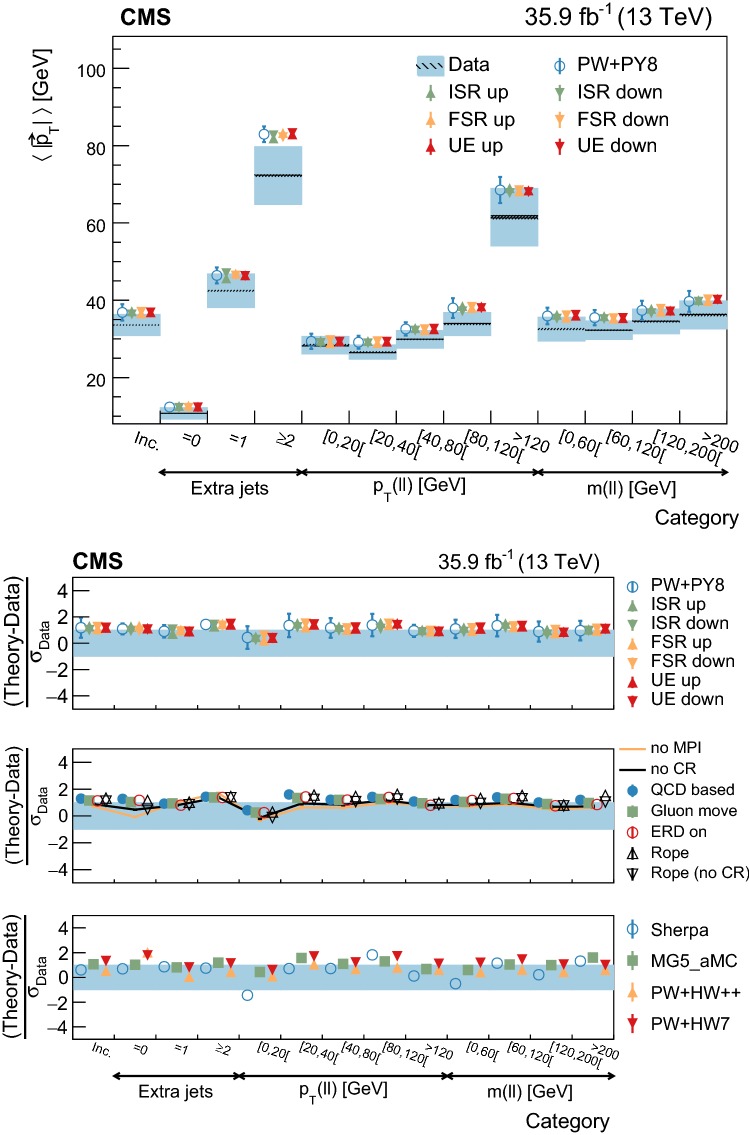
Average $|{\vec {p}}_{\mathrm{T}} |$ in different categories. The conventions of Fig. 14 are used

**Fig. 20:**
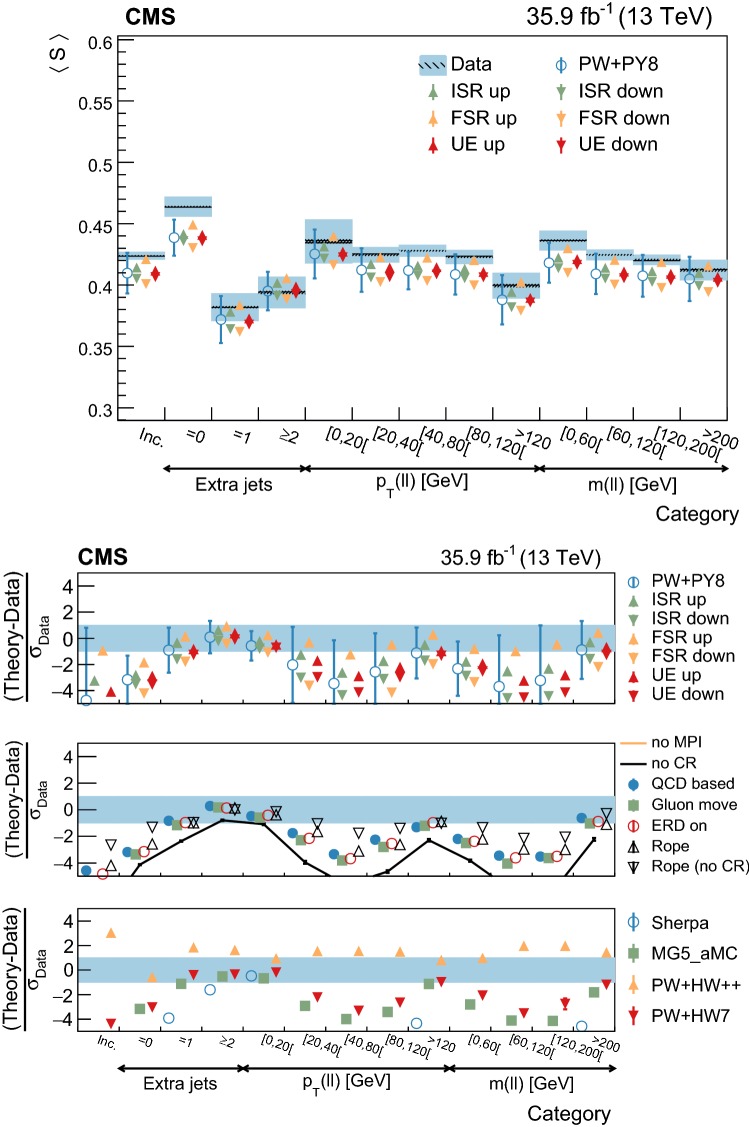
Average sphericity in different categories. The conventions of Fig. 14 are used

**Fig. 21:**
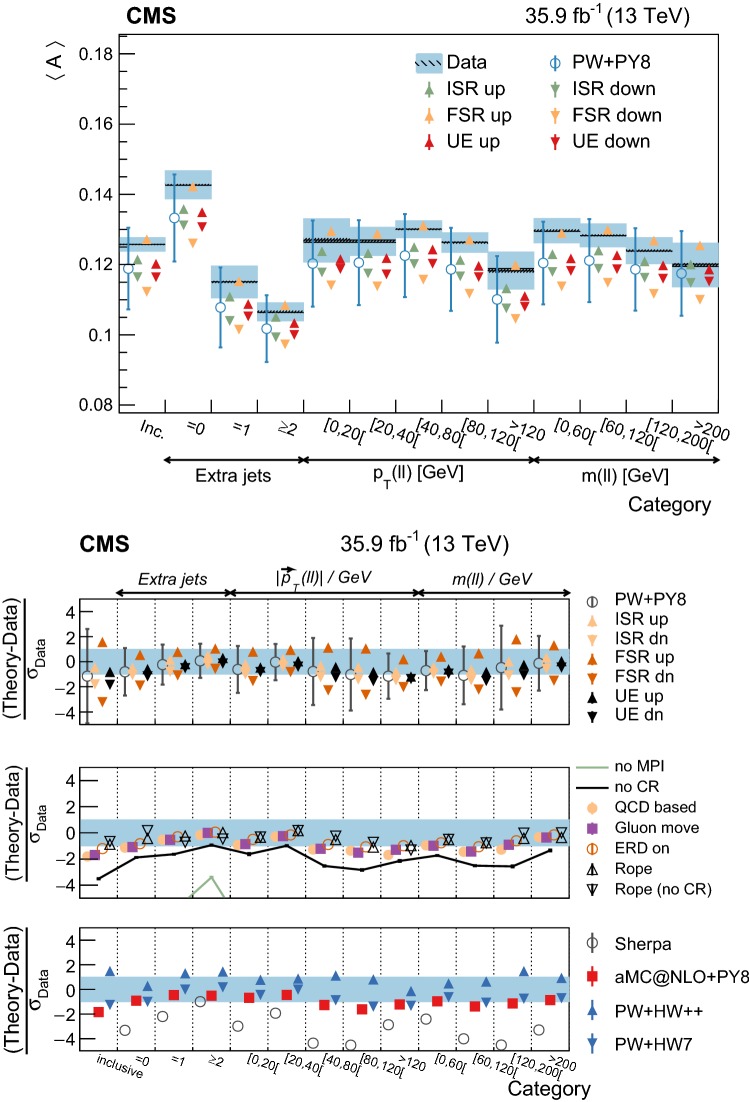
Average aplanarity in different categories. The conventions of Fig. 14 are used

**Fig. 22:**
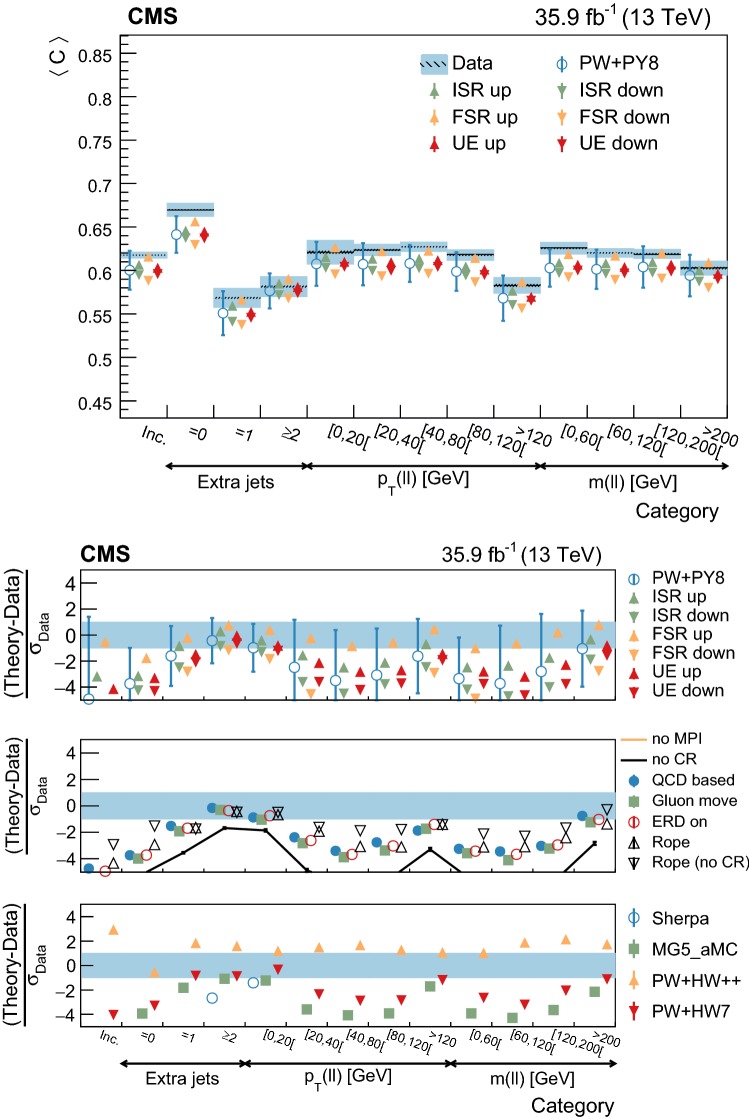
Average *C* in different categories. The conventions of Fig. 14 are used

**Fig. 23:**
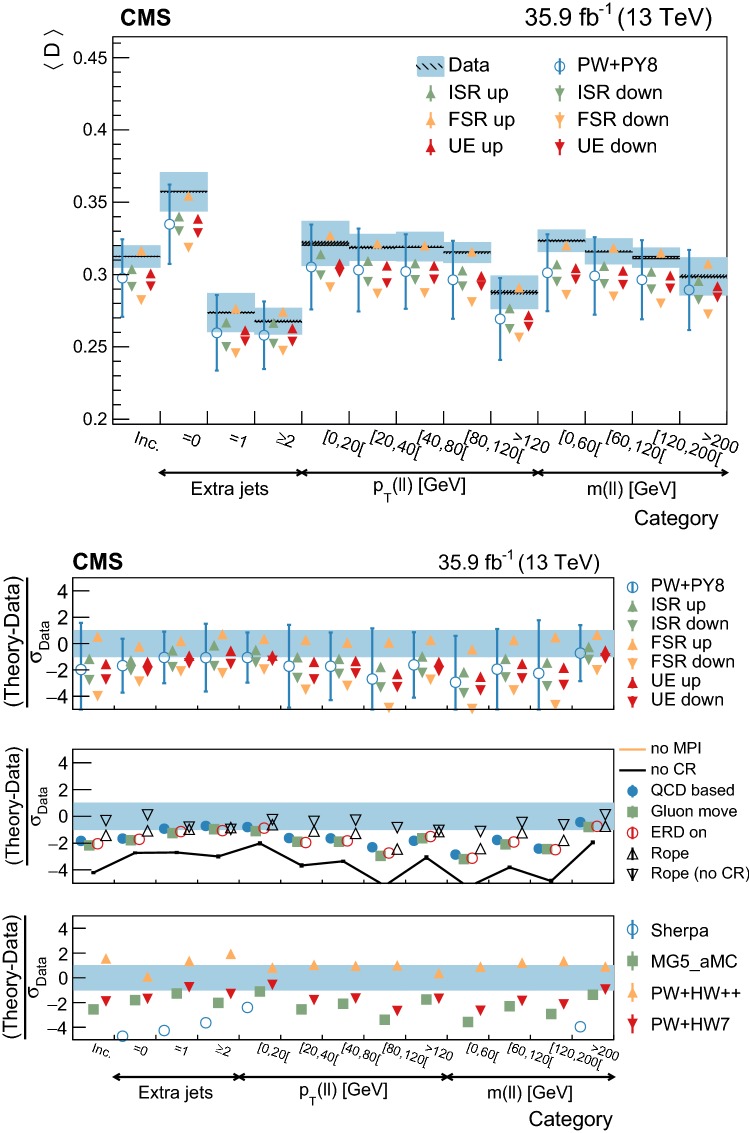
Average *D* in different categories. The conventions of Fig. 14 are used

**Fig. 24:**
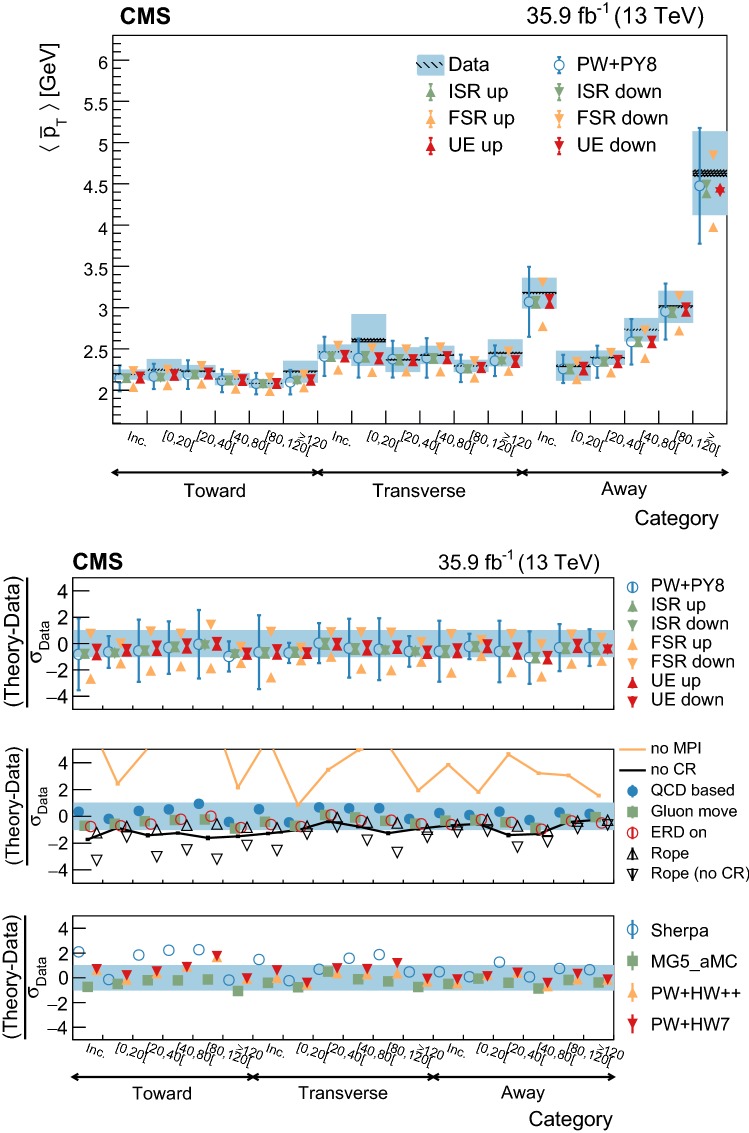
Average $\overline{p_{\mathrm{T}}}$ in different $p_{\mathrm{T}} (\ell \ell )$ categories. The conventions of Fig. 14 are used

**Fig. 25:**
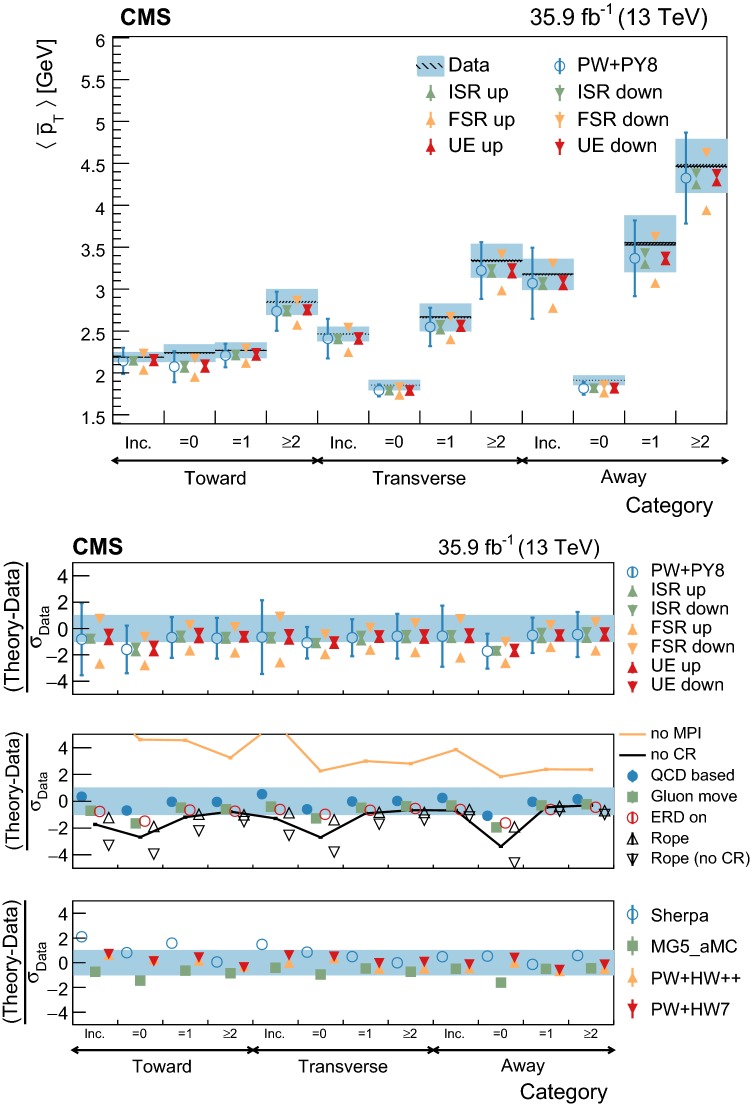
Average $\overline{p_{\mathrm{T}}}$ in different jet multiplicity categories. The conventions of Fig. 14 are used

The differential cross sections as functions of different observables are measured in different event categories introduced in Sect. 5. We report the profile, i.e., the average of the measured differential cross sections in different event categories, and compare it to the expectations from the different simulation setups. Figures 14, 15, 16, 17, 18, 19, 20, 21, 22 and 23 summarize the results obtained. Additional results for $\overline{p_{\mathrm{T}}}$ , profiled in different categories of $p_{\mathrm{T}} (\ell \ell )$ and/or jet multiplicity, are shown in Figs. 24 and 25, respectively. In all figures, the pull of the simulation distributions with respect to data, defined as the difference between the model and the data divided by the total uncertainty, is used to quantify the level of agreement.

The average charged-particle multiplicity and the average of the momentum flux observables vary significantly when extra jets are found in the event or for higher $p_{\mathrm{T}} (\ell \ell )$ values. The same set of variables varies very slowly as a function of $m(\ell \ell )$. Event shape variables are mostly affected by the presence of extra jets in the event, while varying slowly as a function of $p_{\mathrm{T}} (\ell \ell )$ or $m(\ell \ell )$. The average sphericity increases significantly when no extra jets are present in the event showing that the UE is slightly more isotropic in these events. A noticeable change is also observed for the other event shape variables in the same categories.

For all observables, the MPI contribution is crucial: most of the pulls are observed to be larger than 5 when MPI is switched off in the simulation. Color reconnection effects are on the other hand more subtle and are more relevant for $\overline{p_{\mathrm{T}}}$ , specifically when no additional jet is present in the event. This is illustrated by the fact that the pulls of the setup without CR are larger for events belonging to these categories. Event shape variables also show sensitivity to CR. All other variations of the UE and CR models tested yield smaller variations of the pulls, compared to the ones discussed.

Although a high pull value of the Pw+Py8 simulation is obtained for several categories, when different theory variations are taken into account, the envelope encompasses the data. The variations of $\alpha _S ^{\mathrm{FSR}}(M_\mathrm {Z})$ and $\alpha _S ^{\mathrm{ISR}}(M_\mathrm {Z})$ account for the largest contribution to this envelope. As already noted in the previous section, the Pw+Hw++, Pw+Hw7, and sherpa models tend to be in worse agreement with data than Pw+Py8, indicating that further tuning of the first two is needed.

### Sensitivity to the choice of $\alpha _S$ in the parton shower

**Fig. 26:**
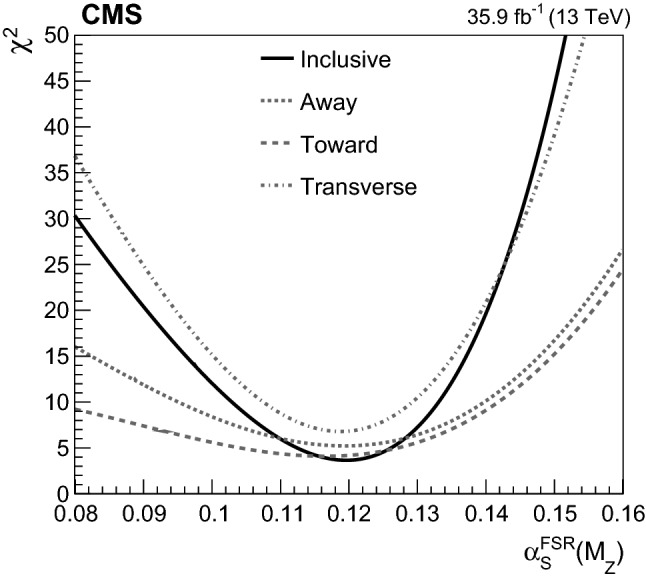
Scan of the $\chi ^2$ as a function of the value of $\alpha _S ^{\mathrm{FSR}}(M_\mathrm {Z})$ employed in the Pw+Py8 simulation, when the inclusive $\overline{p_{\mathrm{T}}}$ or the $\overline{p_{\mathrm{T}}}$ distribution measured in different regions is used. The curves result from a fourth-order polynomial interpolation between the simulated $\alpha _S ^{\mathrm{FSR}}(M_\mathrm {Z})$ points

The sensitivity of these results to the choice of $\alpha _S (M_\mathrm {Z})$ in the parton shower is tested by performing a scan of the $\chi ^2$ value defined by Eq. (3), as a function of $\alpha _S ^{\mathrm{ISR}}(M_\mathrm {Z})$ or $\alpha _S ^{\mathrm{FSR}}(M_\mathrm {Z})$. The $\chi ^2$ is scanned fixing all the other parameters of the generator. A more complete treatment could only be achieved with a fully tuned UE, which lies beyond the scope of this paper. While no sensitivity is found to $\alpha _S ^{\mathrm{ISR}}(M_\mathrm {Z})$, most observables are influenced by the choice of $\alpha _S ^{\mathrm{FSR}}(M_\mathrm {Z})$. The most sensitive variable is found to be $\overline{p_{\mathrm{T}}}$ and the corresponding variation of the $\chi ^2$ function is reported in Fig. 26. A polynomial interpolation is used to determine the minimum of the scan (best fit), and the points at which the $\chi ^2$ function increases by one unit are used to derive the 68% confidence interval (CI). The degree of the polynomial is selected by a stepwise regression based on an F-test statistics [72]. A value of $\alpha _S ^{\mathrm{FSR}}(M_\mathrm {Z})=0.120\pm 0.006$ is obtained, which is lower than the one assumed in the Monash tune [73] and used in the CUETP8M2T4 tune. The value obtained is compatible with the one obtained from the differential cross sections measured as a function of $\overline{p_{\mathrm{T}}}$ in different $p_{\mathrm{T}} (\ell \ell )$ regions or in events with different additional jet multiplicities. Table 4 summarizes the results obtained. From the inclusive results, we conclude that the range of the energy scale that corresponds to the 5% uncertainty attained in the determination of $\alpha _S ^{\mathrm{FSR}}(M_\mathrm {Z})$ can be approximated by a $[\sqrt{2},1/\sqrt{2}]$ variation, improving considerably over the canonical [2, 0.5] scale variations.

**Table 4:** The first rows give the best fit values for $\alpha _S ^{\mathrm{FSR}}$ for the Pw+Py8 setup, obtained from the inclusive distribution of different observables and the corresponding 68 and 95% confidence intervals. The last two rows give the preferred value of the renormalization scale in units of $M_\mathrm {Z}$, and the associated $\pm 1 \sigma$ interval that can be used as an estimate of its variation to encompass the differences between data and the Pw+Py8 setup

$p_{\mathrm{T}} (\ell \ell )$ region	Inclusive	Away	Toward	Transverse
Best fit $\alpha _S ^{\mathrm{FSR}}(M_\mathrm {Z})$	0.120	0.119	0.116	0.119
68% CI	[− 0.006, $+$ 0.006]	[− 0.011, $+$ 0.010]	[− 0.013, $+$ 0.011]	[− 0.006, $+$ 0.006]
95% CI	[− 0.013, $+$ 0.011]	[− 0.022, $+$ 0.019]	[− 0.030, $+$ 0.021]	[− 0.013, $+$ 0.012]
$\mu _\mathrm{R}/M_\mathrm {Z}$	2.3	2.4	2.9	2.4
68% CI	[1.7, 3.3]	[1.4, 4.9]	[1.6, 7.4]	[1.7, 3.5]

## Summary

The first measurement of the underlying event (UE) activity in ${\mathrm {t}\overline{\mathrm {t}}}$ dilepton events produced in hadron colliders has been reported. The measurement makes use of $\sqrt{s}=13~\text {Te}\text {V}$ proton-proton collision data collected by the CMS experiment in 2016, and corresponding to 35.9$~\text {fb}^{-1}$. Using particle-flow reconstruction, the contribution from the UE has been isolated by removing charged particles associated with the decay products of the ${\mathrm {t}\overline{\mathrm {t}}}$ event candidates as well as with pileup interactions from the set of reconstructed charged particles per event. The measurements performed are expected to be valid for other ${\mathrm {t}\overline{\mathrm {t}}}$ final states, and can be used as a reference for complementary studies, e.g., of how different color reconnection (CR) models compare to data in the description of the jets from $\mathrm {W}\rightarrow \mathrm {q} \overline{\mathrm {q}} '$ decays. The chosen observables and categories enhance the sensitivity to the modeling of multiparton interactions (MPI), CR and the choice of strong coupling parameter at the mass of $\mathrm {Z}$ boson ($\alpha _S ^{\mathrm{FSR}}(M_\mathrm {Z})$) in the pythia8 parton shower Monte Carlo simulation. These parameters have significant impact on the modeling of ${\mathrm {t}\overline{\mathrm {t}}}$ production at the LHC. In particular, the compatibility of the data with different choices of the $\alpha _S ^{\mathrm{FSR}}(M_\mathrm {Z})$ parameter in pythia8 has been quantified, resulting in a lower value than the one considered in Ref. [73].

The majority of the distributions analyzed indicate a fair agreement between the data and the powheg +pythia8 setup with the CUETP8M2T4 tune [18], but disfavor the setups in which MPI and CR are switched off, or in which $\alpha _S ^{\mathrm{FSR}}(M_\mathrm {Z})$ is increased. The data also disfavor the default configurations in powheg +herwig++, powheg +herwig7, and sherpa. It has been furthermore verified that, as expected, the choice of the next-to-leading-order matrix-element generator does not impact significantly the expected characteristics of the UE by comparing predictions from powheg and MadGraph 5_amc@nlo, both interfaced with pythia8.

The present results test the hypothesis of universality in UE at an energy scale typically higher than the ones at which models have been studied. The UE model is tested up to a scale of two times the top quark mass, and the measurements in categories of dilepton invariant mass indicate that it should be valid at even higher scales. In addition, they can be used to improve the assessment of systematic uncertainties in future top quark analyses. The results obtained in this study show that a value of $\alpha _S ^{\mathrm{FSR}}(M_\mathrm {Z})=0.120\pm 0.006$ is consistent with the data. The corresponding uncertainties translate to a variation of the renormalization scale by a factor of $\sqrt{2}$.

## Data Availability

This manuscript has no associated data or the data will not be deposited. [Authors’ comment: Release and preservation of data used by the CMS Collaboration as the basis for publications is guided by the CMS policy as written in its document "CMS data preservation, re-use and open access policy" (https://cms-docdb.cern.ch/cgibin/PublicDocDB/RetrieveFile?docid=6032&filename=CMSDataPolicyV1.2.pdf&version=2).].

## A Appendix: Variations of the powheg +pythia8 setup

See Table 5.

**Table 5:** Variations of the Pw+Py8 setup used for the comparison with the measurements. The values changed with respect to the CUETP8M2T4 tune are given in the columns corresponding to each model. Further details on parameters or specificities of the models can be found in Refs. [4, 5, 17, 32, 32–34, 73]. For the Rope hadronization model two variations are considered: one with no CR and the other with the default CR model. The settings for the former are denoted in parenthesis in the last column

Parameter	Pw+Py8 simulation setups									
	CUETP8M2T4	Extreme variations		Fine grain variations						
				MPI/CR	Parton shower scale		CR including ${\mathrm {t}\overline{\mathrm {t}}}$			
		no	no	UE	ISR	FSR	ERD	QCD	Gluon	Rope (no CR)
		MPI	CR	up/down	up/down	up/down	on	based [32]	move [5]	[33, 34]
PartonLevel										
MPI	on	off								
SpaceShower										
renormMultFac	1.0				4/0.25					
alphaSvalue	0.1108									
TimeShower										
renormMultFac	1.0					4/0.25				
alphaSvalue	0.1365									
MultipartonInteractions										
pT0Ref	2.2			2.20/2.128				2.174	2.3	
ecmPow	0.2521							0.2521		
expPow	1.6			1.711/1.562				1.312	1.35	
ColorReconnection										
reconnect	on		off							(off)
range	6.59			6.5/8.7						
mode	0							1	2	
junctionCorrection								0.1222		
timeDilationPar								15.86		
m0								1.204		
flipMode									0	
m2Lambda									1.89	
fracGluon									1	
dLambdaCut									0	
PartonVertex										
setVertex										on
Ropewalk										
RopeHadronization										on
doShoving										on
doFlavour										on
PartonLevel										
earlyResDec	off						on	on	on	on
